# The computational neurology of movement under active inference

**DOI:** 10.1093/brain/awab085

**Published:** 2021-03-11

**Authors:** Thomas Parr, Jakub Limanowski, Vishal Rawji, Karl Friston

**Affiliations:** 1 Wellcome Centre for Human Neuroimaging, Queen Square Institute of Neurology, University College London, London WC1N 3BG, UK; 2 Faculty of Psychology and Center for Tactile Internet with Human-in-the-Loop, Technische Universität Dresden, Dresden, Germany; 3 Department of Clinical and Movement Neurosciences, Queen Square Institute of Neurology, University College London, London WC1N 3BG, UK

**Keywords:** neurology, active inference, movement, Bayesian, planning

## Abstract

We propose a computational neurology of movement based on the convergence of theoretical neurobiology and clinical neurology. A significant development in the former is the idea that we can frame brain function as a process of (active) inference, in which the nervous system makes predictions about its sensory data. These predictions depend upon an implicit predictive (generative) model used by the brain. This means neural dynamics can be framed as generating actions to ensure sensations are consistent with these predictions—and adjusting predictions when they are not. We illustrate the significance of this formulation for clinical neurology by simulating a clinical examination of the motor system using an upper limb coordination task. Specifically, we show how tendon reflexes emerge naturally under the right kind of generative model. Through simulated perturbations, pertaining to prior probabilities of this model’s variables, we illustrate the emergence of hyperreflexia and pendular reflexes, reminiscent of neurological lesions in the corticospinal tract and cerebellum. We then turn to the computational lesions causing hypokinesia and deficits of coordination. This *in silico* lesion-deficit analysis provides an opportunity to revisit classic neurological dichotomies (e.g. pyramidal versus extrapyramidal systems) from the perspective of modern approaches to theoretical neurobiology—and our understanding of the neurocomputational architecture of movement control based on first principles.

## Teaser

Parr *et al.* report a series of numerical simulations—based on Bayesian message passing—of healthy and pathological motor control. They perform an *in silico* lesion-deficit analysis and demonstrate how the sorts of syndromes observed during a clinical neurological examination emerge from pathological computation.

## Introduction

Much of our understanding of neurobiology rests upon observations from clinical neurology. The classification of symptoms and signs according to different sorts of lesion—and the implied distinctions between the systems these disrupt—underwrite a modern understanding of the function of the nervous system.^1^^,^^2^ One of the outstanding challenges for modern neuroscience is to supplement these observations with formal (computational) models and to establish their theoretical foundations. In what follows, we revisit some fundamental ideas in clinical neurology from the perspective of a formal approach to neurobiology. Our aim was to determine whether there is a clear analogy between the distinctions that arise through purely theoretical considerations and those that have been established through clinical observation.

The utility of this approach has previously been unpacked in the setting of functional neurological disorders.^3^ The importance of a computational understanding of ‘functional’ pathology is underwritten by the difficulty in identifying gross structural abnormalities in these patients. Edwards *et al.*^3^ illustrated—using a predictive coding formalism—that computational pathology manifesting at a synaptic level affords a plausible explanation for functional signs and symptoms. In addition to furthering our understanding of these disorders, this identifies (broadly) the kinds of therapy that could be developed to treat them. Crucially, as this approach is based on a physiologically grounded theory, these therapeutic approaches include those that act to pharmacologically modulate synaptic transmission or involve behavioural therapies designed to target the same synapses through their associated computational role. Here, we argue that the benefits outlined by Edwards *et al.*^3^ may be usefully extended and unified with other neurological subfields. In other words, a functional understanding of neurological syndromes should not be restricted to those patients for whom no structural lesion has been identified. As a starting point, it is important to find the points of connection between classical neurological and computational framings of brain function.

For this challenge to be met, we examined some of the important distinctions made between the types of clinical signs and ask whether these map on to the distinctions that arise from theoretical neurobiology. To ensure the relevance of this in clinical settings, we focused on two sorts of behaviour commonly elicited in neurological examination that vary with different pathologies. The first was an examination of tendon stretch reflexes. This is an important part of a neurological examination in which tapping on a tendon elicits a reflexive movement at that joint.^4^ The amplitude, speed and shape of the response is indicative of certain kinds of pathology. Notably, damage to the corticospinal tract—which provides cortical modulation of spinal reflex circuits—leads to ‘brisk’ reflexes with large amplitude. Cerebellar lesions, on the other hand, lead to oscillatory or ‘pendular’ reflexes.

The other domain we considered was the examination of coordination, as assessed through the common ‘finger-nose’ test, in which patients are asked to alternate between touching their nose and then the finger of the examining clinician. The clinician moves their finger to alternative locations as the patient continues. This is useful in identifying ataxias—often due to cerebellar lesions.^5^ While not the primary method of assessment, performance of this test may be difficult for those with parkinsonian phenotypes where movement initiation is impaired. Our focus on these tests rested upon their established ability to disambiguate between different kinds of clinical syndrome. In addition, they provide an important test of construct validity for a hypothetical computational lesion, as their consequences must be consistent with clinical pathology in different domains. A theoretically motivated cerebellar lesion is clearly a poor hypothesis if it induces pendular reflexes but no ataxia and vice versa. Finally, an appeal to clinical signs that localize anatomical lesions offers a test of a computational architecture for motor control. The connectivity of lesioned areas must be consistent with that established through neurological anatomy.

Broadly, the theoretical distinctions of interest here concern the difference between spatial and temporal precision, continuous and categorical inference and inference about states of the world and planning as (active) inference. The relevant clinical distinctions are between pyramidal and extrapyramidal, cerebellar and subcortical and motor and executive syndromes. In setting out the relationship between these, we asked whether modern theoretical neurobiology endorses these clinical categories and, if so, whether we could use the wealth of anatomical knowledge associated with neurological constructs to constrain accounts of brain function in terms of inferential message passing. The purpose of this paper was to find a mapping between the Bayesian message passing that could be used to generate movements and the neuroanatomy of motor control.

The challenge of unifying structural and functional accounts of brain dysfunction calls for a model that not only predicts behaviour but is grounded in the structural anatomy of the brain. The benefit of a forward model—that makes predictions at the level of behaviour—is 3-fold. First, it is useful to have a common framework in which to understand functional changes in the brain following a variety of pathological processes. We focused on the framework afforded by Bayesian accounts of brain function—which assume that the brain employs an internal model to draw inferences about the causes of sensory data. The flexibility of an inferential formulation of brain function is evident in accounts of neurological phenomena as diverse as synucleinopathic visual hallucinations,^6^ alien limb syndrome in cortico-basal degeneration^7^ and tic disorders.^8^

Second, it is useful to know what is and is not a plausible explanation for a given clinical sign. A computational model provides a simple means of assessing this. By inducing a hypothetical lesion and simulating the results, we see whether or not that hypothesis could account for observed pathology. We provide an example of this in the 'Synthetic behaviour' section in relation to tremor in Parkinson’s disease. In brief—consistent with previous arguments^9^—we found that tremor is not explained by a lesion of the computational homologue of the substantia nigra pars compacta. Instead, this may be a downstream consequence of (pathological or therapeutic) perturbations to one aspect of brain function for distant parts of the network (*cf.* functional diaschisis^10-12^).

Finally, forward models aid the non-invasive quantification of pathology. Using standard model inversion schemes,^13^ it is possible to infer the parameter values that best explain observed behaviour. The advantages of doing so include the ability to track disease progression and therapeutic responses quantitatively. This is useful both in the therapeutic setting and in clinical trials, where such measures could form useful outcome measures. Alternatively, quantitative phenotyping is useful in identifying candidates for trials or treatment options. The benefits of this sort of approach are evident in quantitative (genomic) phenotyping in cancer research.^14^

The key theoretical contributions we offer in this paper are as follows. First, we set out a generative model whose motoric solutions include the kinds of behaviours used to assess neurological function in a clinical setting. Second, this generative model uses a hierarchical form where each level prescribes short trajectories at the level below—consistent with the idea of motor chunking,^15^ the idea that the single motor elements of action sequences can be grouped into units of behaviour, over multiple timescales. Third, the synthetic lesion-deficit analysis provided here goes beyond previous mixed models based upon active inference^16^ and provides a means to map message passing to known neurological anatomy.

## Active inference

We begin by outlining active inference; a ‘first principles’ account of behaviour.^17^ We do so to highlight the key dichotomies that are implied. The central idea is that the brain’s dynamics can be framed in terms of an implicit internal (generative) model as if it were drawing inferences about the outside world.^18^ Under active inference, perception and action are framed as processes that try to reconcile discrepancies between predictions of the generative model and the world,^19^ either by changing beliefs (perception) or changing the world (action). [It is worth acknowledging a tension between some of the technical terms we use, and the lay meaning of these words. When we refer to a ‘belief’, we mean this in the Bayesian sense (i.e. Bayesian belief updating or propagation). Here, a belief is simply a probability distribution. This may be represented by the activity of a neural population—not a propositional belief in the folk psychology sense.] As such, the key to understanding healthy or pathological behaviour is in finding the generative model that the brain is implicitly using.^20^ In the context of movement, there are two parts to this generative model.^16^ One that deals with alternative movements that could be made (e.g. ‘Do I reach to the left or the right?’), and one that details the sensory consequences anticipated during the execution of that movement (e.g. ‘If I reach to the left, what does this mean for proprioceptive input at my shoulder joint?’).

The role of a generative model is to predict sensory data (*y*). The quality of predictions under a model, relative to observed data, may be quantified in terms of ‘evidence’—the probability that observed data would have been generated by this model. The (log) evidence can be written in terms of the joint density of hidden (unobserved) variables [*υ* = (*x*, *v*, *s*, *π*)], data generated by the generative model and the posterior probability of these hidden variables having observed these data:

(1) $$\begin{array}{c} \ln p(y)=\ln\;p(y,\upsilon )-\ln\;p(\upsilon |y)\\ =E_{p(\upsilon |y)}[\ln p(y,\upsilon )-\ln\;p(\upsilon |y)]\\ \geq E_{q(\upsilon )}[\ln p(y,\upsilon )-\ln\;q(\upsilon )]\\ =\ln\;p(y)-D_{KL}[q(\upsilon )||p(\upsilon |y)] \end{array}$$

The first line here illustrates the decomposition of the log evidence into a joint probability and a posterior probability via Bayes’ rule. The second line (arbitrarily) introduces an expectation (i.e. average) with respect to the posterior probability. Given that the left-hand side is not explicitly a function of the hidden variables, this expectation does nothing to this expression. However, it does let us construct a lower bound on the evidence by relaxing the constraint that we use the exact posterior density.^21^^,^^22^ This is useful, as it is often difficult to compute the exact posterior. The third line expresses this bound by substituting an arbitrary distribution (*q*) for the posterior. The rearrangement in the final line (which rests upon factorizing the joint distribution into the evidence and posterior) shows that the Kullback–Leibler divergence (which quantifies how different two distributions are from one another) between our approximate posterior and the exact posterior density quantifies the difference between the model evidence and its lower bound (sometimes referred to as an ELBO or a negative free energy).

The insight from Equation 1 is that, if we wish to act upon the environment to obtain those data that maximize the evidence for a model, it is sufficient to seek those data that maximize a lower bound, as long as the divergence between our approximate posterior and the exact posterior is small. This lets us summarize active inference as follows:

(2) $$\begin{array}{c} a=\underset{a}{\arg\;\max}\;E_{q(\upsilon )}[\ln\;p(y(a)|\upsilon )]\\ q(\upsilon )=\underset{q(\upsilon )}{\arg\;\max}\;\{E_{q(\upsilon )}[\ln\;p(y(a)|\upsilon )]-D_{KL}[q(\upsilon )||p(\upsilon )]\} \end{array}$$

The first line says that we choose the actions (*a*) that lead to the most probable data under current beliefs. These data are expressed as a function of action [*y*(*a*)] to emphasize that action changes the outside world in such a way that we receive new sensory data. The second line revises beliefs, such that they render the data more probable (while not moving too far from prior beliefs). The reason the Kullback–Leibler divergence in the second line does not appear in the first is that this term does not depend upon sensory data and so is not changed by action. An important aspect of Equation 2 is that the optimal action given a sensory input depends upon the prior beliefs that characterize a generative model. The importance of this relates to a set of results known as the complete class theorems.^23^^,^^24^ These state that any behaviour (or statistical decision function) is Bayes optimal under the right set of priors. In one sense, this says that Equation 2 is trivial, in that there are a set of prior beliefs that renders it true for any given observed behaviour. In another sense, this provides a useful way of articulating the challenge before us in characterizing healthy or pathological behaviour. It says that the appropriate way to provide this characterization is to find the set of priors under which that behaviour—healthy or pathological—would be Bayes optimal. In other words, we seek to identify the priors that would generate specific sorts of behaviour when Equation 2 is solved for their associated generative model. Ultimately, one might hope that this will inform diagnosis and treatment of motor maladies. The optimization scheme used to solve Equation 2 for the generative model used here is detailed in the Supplementarymaterial.

To apply Equation 2 in a more concrete setting, we must specify the generative model [*p*(*y*, *υ*)] associated with that setting. We will begin with the model that accounts for the generation of continuous proprioceptive and visual data based upon arm movements, which brings us to realm of (bicipital) tendon stretch reflexes.

## Synthetic tendon reflexes

**Figure 1:**
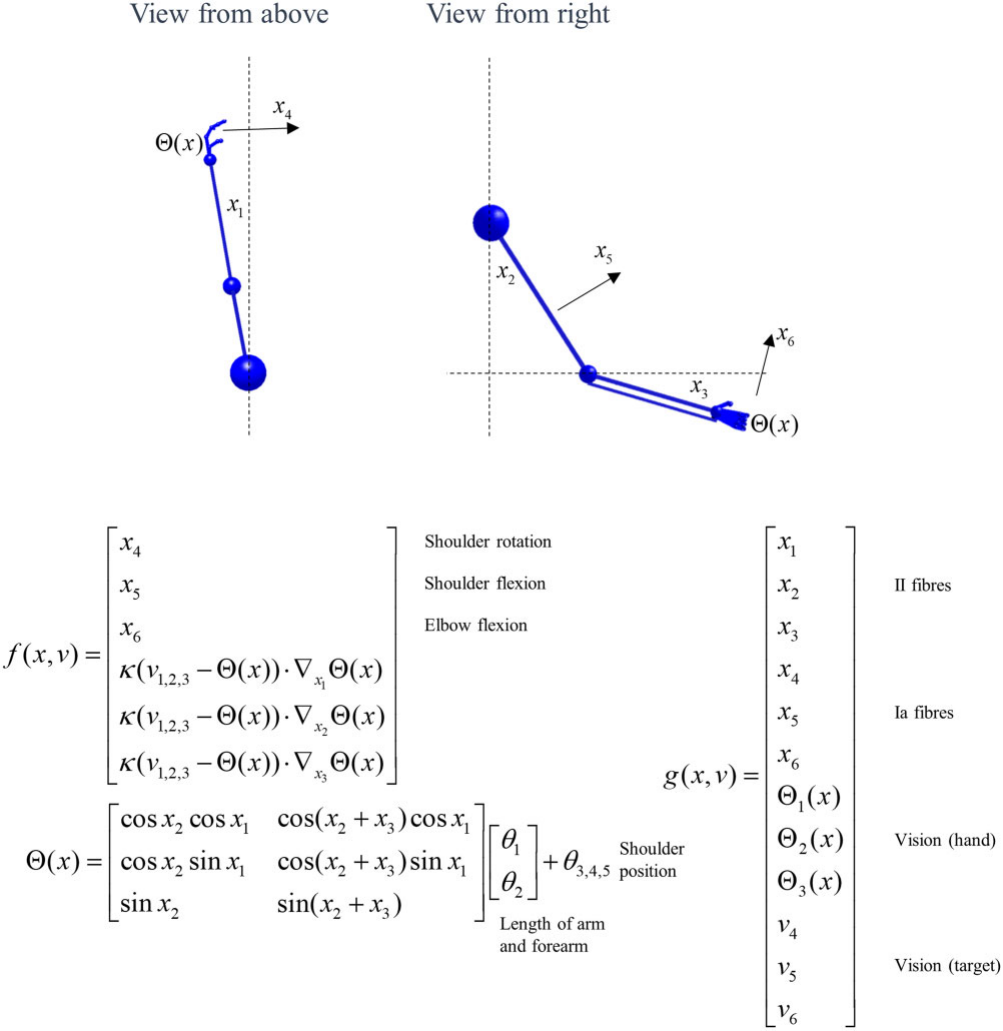
**Continuous generative model of arm movement.** This schematic shows the variables used to define a generative model of arm movements in three dimensions, and the equations that mediate their interactions. The functions *f* and *g* are used to define the expected rate of change of *x*, and the generation of *y*. The first three elements of *x* are the angular positions of the shoulder (rotation and flexion) and the elbow (flexion). The final three are the angular velocities associated with these joints. We have designed this generative model to include the belief that there is a (fictitious) force that is proportional to the distance between the position of some (imaginary) target (*v*_1,2,3_) and the current position of the hand (Θ) in a three dimensional Euclidean frame of reference. The Θ function takes the angular positions of the joints and returns the coordinates of the hand. This depends upon the lengths of the arm and forearm, and the position of the shoulder (*θ*). Sensory data are divided into proprioceptive and visual modalities. The proprioceptive II-fibres report the angular position of the joints (i.e. the ‘stretch’ in the associated tendons), while Ia-fibres report the rate of change of these positions. We have adopted the simplification here that the visual modalities report the position of the hand in Euclidean coordinates. In addition, they specify the colour of each of the three targets (*v*_4,5,6_) in different spatial positions. This model is similar in spirit to that used for (2-dimensional) simulations of handwriting.^26^

The first problem we face is how to generate a healthy tendon reflex. This means we write down the generative model that predicts proprioceptive data from the relevant joints and solve Equation 2 for this model. How do we relate Equation 2 to the idea of a reflex? This equation says we should take action to ensure incoming (proprioceptive) data cohere with their most likely value. Another perspective is that an internal model provides a setpoint for these sensory data, and that any deviations from this point must be corrected by action (i.e. changes in firing of motor neurons). Framed in terms of spinal reflexes, this means proprioceptive signals resulting from changes in muscle length are propagated to the dorsal horn of the spinal cord (the afferent pathway) where they induce changes in firing of motor neurons (the efferent pathway), which restore the muscle length to their set-point.^25^ The set-point may be modulated by corticospinal projections, which influence the relationship between the afferent and efferent pathways. This influence, under active inference, depends upon the form of the implicit generative model. Figure 1 specifies the form of the model. This takes the things we expect to influence sensory data coming from an arm, including the angular positions and velocities of the shoulder and elbow joints, and predicts the sensory input expected under a given configuration. The velocity of the joints is determined by a ‘target’ location, as if the hand is being pulled towards a desired location. This may be seen as a formalization of the ‘equilibrium point hypothesis’^27^ and the more recent ‘passive movement’ paradigms.^28^ The key idea behind these formulations is that a movement may be generated simply by predicting its sensory consequences—as if it were taking place—and using low level reflexes to correct any discrepancies. In other words, if we were to imagine our arm being pulled to a point in space, we can predict the proprioceptive input we would experience and use this to realize the movement. This kind of model is similar to those used in modern robotics to generate movements^29^^,^^30^ and to established approaches in engineering including proportional-integral-derivative control.^31^ Within the same framework, it follows that a discrepancy between proprioceptive input and sensory predictions would also result in impaired voluntary movement; as is seen in the case of severe deafferentation.^32^

**Figure 2:**
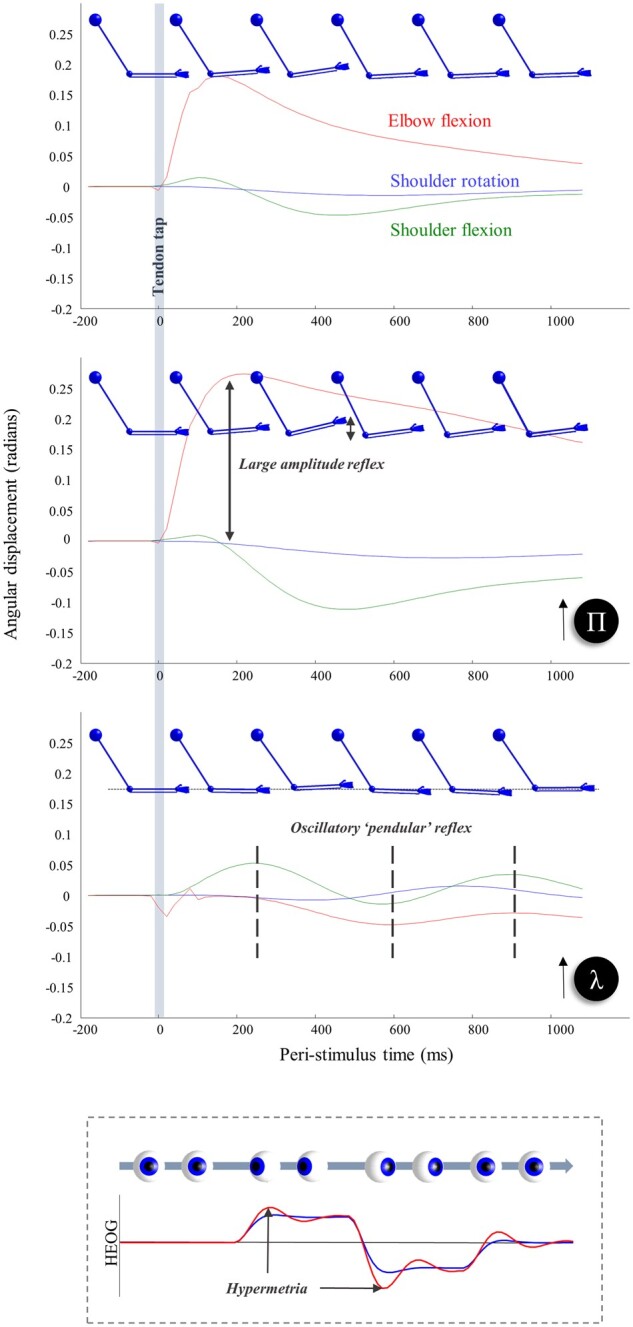
**Synthetic biceps reflex under normal and abnormal precision estimates.** The top three plots show the consequence of briefly stimulating the stretch receptors at the elbow, as if we had tapped on the tendon connecting the biceps muscle to the bones of the forearm. This induces a (proprioceptive) prediction error that is corrected by spinal reflexes. The top plot illustrates this for a ‘healthy’ generative model. The next two plots show what happens if we perturb beliefs about the spatial and temporal components of precision, respectively. Overestimating the former (Π) increases the gain of the prediction error, increasing the speed and amplitude of the reflex. Overestimating the smoothness of random fluctuations (λ) instead induces a smaller amplitude ‘pendular’ reflex where the arm continues to oscillate following the tendon tap. Note that the effects of the tendon tap are not seen only in the elbow joint. There are additionally changes in the shoulder flexion; changes in the rotation of the shoulder are limited, as this is orthogonal to the elbow joint. The bottom plot illustrates the same perturbation for the model of oculomotor behaviour described in Parr and Friston.^33^ In this context, overestimation of the smoothness leads to hypermetric saccades. Compare this (red) to the simulated horizontal electro-oculogram (HEOG) in blue, where the smoothness is correctly estimated. The graphics of the arms are shown to provide some intuition for non-clinical readers as to the subtlety of the signs that might be elicited during a neurological examination. For clinical readers, they illustrate the type of patterns they might be familiar with. While still images are limited in providing a sense of what is happening here, we invite readers to run the demonstration code indicated in the software note to produce an animated version of these graphics. In this setting, it is much easier to appreciate the qualitative differences between each lesion.

The equations in Fig. 1 are obtained through application of Newtonian mechanics and trigonometry. The angular positions of the joints (*x*_1,2,3_) change at a rate specified by their angular velocities (*x*_4,5,6_). The rate of change of the velocities depends upon the product of the moment of inertia (*κ*) and a rotational force. In the ‘real’ world, this force is given by the action (*a*) generated as a consequence of Equation 2. From the perspective of the generative model shown in Fig. 2, the rotational force is an imagined (fictitious) force that acts in a Euclidean frame of reference to shorten the distance between the position of the hand (Θ) and a target position (*v*_1,2,3_). This distance is multiplied by the gradient of Θ with respect to the angular positions to bring this back into angular coordinates. The function Θ—which gives the Euclidean position of the hand as a function of the angular positions of the joints—follows from standard trigonometry. The function *g* maps these variables to the expected data in each modality. In addition, *g* reports the colour of the targets (whose positions are fixed) based upon the *v*_4,5,6_ variables. This gives three numbers that represent the intensity of the shading for each target.

Having set up the continuous part of the model, we are now in a position to explore the influences of some simple perturbations. To illustrate the effects of these, we use a ubiquitous neurological test: eliciting the biceps tendon reflex. This involves using a tendon hammer to tap on the tendon connecting the biceps muscle to the bones of the forearm.^4^ We simulate this by transiently augmenting the input of the type Ia proprioceptive afferents at the elbow, as if the tendon had been suddenly stretched. The first plot in Fig. 2 illustrates the consequences of this in the absence of synthetic lesions. As *in vivo*, this induces a fast, small amplitude flexion of the elbow that then relaxes back to its initial position. The reason for this is that the artificial proprioceptive input carries low evidence under the generative model (that, *a priori*, does not entertain perturbations of this sort); thus, action is induced to restore these data to their most probable values.

To select the most appropriate candidates for synthetic lesions, it is worth explaining in more detail about the notion of ‘precision’.^34^ This is the inverse variance associated with a probability distribution. In engineering and motor control, precision can be regarded as the gain applied to corrective prediction errors. In terms of inference, precision may be thought of as the confidence of a belief, as opposed to its content, and can have debilitating consequences when it is not estimated accurately. For a dynamic model, of the sort employed here, precision may be factorized into two components (see the Supplementary material for details):

(3) $$\tilde{\Pi }=\Pi ⊗S(\lambda )$$

Loosely speaking, these may be thought of as spatial (Π) and temporal [*S*(*λ*)] components, in the sense that the former accounts for the inverse covariance of the positions of the (*x*, *y* or *v*) variables, while the latter determines the smoothness of random fluctuations (i.e. the correlations between position, velocity and subsequent temporal derivatives). The point of this decomposition is that, for a biological system, these fluctuations are not really ‘random’ but are generated by dynamical systems operating over a faster timescale than that considered by the controllable dynamics or kinetics of the generative model.^35^ This means that these fluctuations have a structure over time (e.g. serial correlations or smoothness) that cannot be neglected. The use of generalized coordinates of motion and the definition of the generalized precision matrix of Equation 3 ensures we take account of this temporal structure. The *λ* parameter is a parameter of the autocorrelation function, evaluated at zero lag, and can be thought of as a measure of the ‘smoothness’ of the fluctuations.

Accurate estimation of precision is vital in the context of movement in that descending predictions of the sensory consequences of movement must predict not only the expected value of these sensory signals but the dispersion anticipated around this expectation.^36^ This underwrites the notion of sensory attenuation^37^; attending away from sensory data during self-generated movement. On this view, descending predictions of Π should be attenuated by descending corticospinal fibres.^38^ To show what happens in the absence of this attenuation, we simulated a tendon reflex with a generative model that attributes too much precision to sensory data; i.e. as if we had induced a lesion somewhere between the motor cortex and spinal motoneurons—to induce a failure of sensory attenuation. Compare the top plot in Fig. 2, which shows the ‘healthy’ response, with the middle plot showing the exaggerated ‘brisk’ reflex^39^^,^^40^ following a failure to attenuate predictions about the precision of these data.

The hypothesis that arises from this is that the corticospinal tract normally attenuates the gain (i.e. precision) of the synapses mediating spinal stretch reflexes and that this gain is abnormally high in the absence of corticospinal attenuation. To assess the validity of this hypothesis, one should consider other pathological processes that lead to brisk reflexes with an intact corticospinal tract. Our hypothesis implies that such processes should also preclude attenuation of reflexive gain. This seems to be borne out by an autoimmune condition known as stiff-person syndrome—which causes hyperreflexia in the presence of a structurally intact corticospinal tract.^41^^,^^42^ This is due to autoantibodies against an enzyme called glutamate decarboxylase (GAD). Physiological measurements that hint at excessive synaptic gain include the normal motor unit activity on electromyography during muscle spasms.^43^ One interpretation of these responses is that normally subthreshold sensory inputs evoke what would be normal efferent aspects of a reflex. In other words, the gain of the translation from the afferent to efferent input is turned up.

GAD is an enzyme found (not exclusively) in the dorsal horn of the spinal cord^44^ and is involved in the synthesis of the inhibitory neurotransmitter GABA. There is evidence of disruption of the associated spinal circuits in stiff-person syndrome.^45^^,^^46^ This is interesting in that the disruption leading to hyperreflexia involves loss of inhibitory neurotransmitters in the region of the same synapses whose gain is attenuated by the corticospinal system. The implication here is that both corticospinal disconnection and anti-GAD disease fail to suppress exuberant responses of efferent in response to afferent arms of a reflex. Computationally speaking, this is a failure to attenuate precision.

Interestingly, stiff-person syndrome is associated with startle reflexes that can be evoked by sensory modalities other than proprioception; including audition and somatosensation.^42^ This implies the mechanisms behind a stretch reflex, normally observed following anatomically localized lesions, may be more broadly expressed. While vascular events that disrupt the pyramidal tract do not give rise to multimodal startle responses, autoimmune processes do not need to respect these anatomical boundaries.

The bottom plot in Fig. 2 shows the consequence of overestimating the smoothness of fluctuations (i.e. the temporal component of the precision of generalized motion). The consequence of this is a pendular reflex, inducing oscillatory movements at the elbow and shoulder. This sort of response is characteristic of cerebellar lesions,^47^^,^^48^ although normally demonstrated at the knee, and implicates the cerebellum in estimation of these temporal correlations. The inset below the bottom plot shows the result of the same lesion applied to the oculomotor model described by Parr and Friston^33^ illustrating the hypermetric saccades and oscillatory corrective movements that result from overestimation of smoothness in this domain. Similar effects are seen in cerebellar lesions.^49–51^ The idea that the cerebellum may be engaged in optimizing beliefs about the temporal component of precision harmonizes with ideas about the role of this structure in the precise timing of responses.^52^^,^^53^ Specifically, the higher temporal embedding order allows for more precise beliefs about the local trajectory of continuous variables due to the non-zero autocorrelations that come along with this. This facilitates local predictions about ‘when’ something is likely to change. Notably, it is this temporal embedding order that has previously been exploited to account for sensorimotor delays within a predictive coding (motor) scheme.^54^

At this point, it is worth reflecting on where this approach is situated compared to other perspectives on motor control. Specifically, it is important to acknowledge that some argue in favour of separable, but interacting, processes that mediate state estimation and motor control. This implies that, in addition to a forward model of the kind employed here, there is an ‘inverse’ model that specifically deals with computing a motor command that would fulfil a desired goal.^55^^,^^56^ The approach we pursue here is more aligned with pure forward modelling approaches that generate predicted proprioceptive data and use spinal reflex arcs to resolve discrepancies between predicted and observed sensory inputs,^57^ which renders inverse modelling redundant. A feature common to both perspectives is the notion of optimization.^58^ Each relies upon a function (or functional, under active inference) that must be minimized or maximized. While a full deconstruction of these two approaches requires a paper of its own, some of the salient points are highlighted in the Supplementary material. This is not intended as a refutation of previous accounts of reflexes but as a formalization in terms of the same sort of message passing used in more ‘cognitive’ operations.

In this section, we have highlighted the first dichotomy that emerges from specifying the kind of generative model required for arm movements. This is the distinction between spatial and temporal contributions to the precision of fluctuations. Interestingly, at least in the context of tendon reflexes, these appear to mirror the distinction between cerebellar and corticospinal syndromes. Our aim in the next section is 2-fold. First, by moving on to the next (coordination) stage of a neurological examination, we have an opportunity to assess whether the synthetic cerebellar and corticospinal homologues introduced here generalize beyond the domain of reflexes. Second, we introduce our next dichotomy: inference about continuous or categorical variables, capturing the distinction between execution and planning.

## Hierarchy and planning

**Figure 3:**
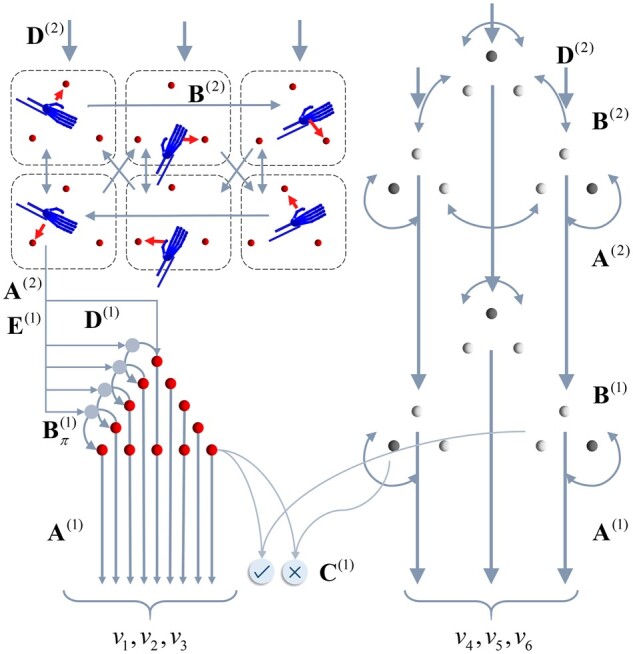
**Discrete generative model for movement planning.** This schematic illustrates the hierarchical discrete state-space generative model that sits above the continuous model shown in Fig. 1. This model generates the hidden causes (*v*) that are the (imaginary) attracting points and the target locations from the perspective of the continuous model, which effectively induce movement. The discrete (categorical) causes that generate these come in two forms: the alternative attracting points (red spheres) that act as equilibrium points, and which of the three possible target locations is currently specified. These causes are themselves generated by states at a higher level. At the highest level (upper left) we have a set of alternative combinations of trajectories. Each of these is defined in terms of which vertex of a triangle (i.e. target location) is at the start and end of that trajectory. There are three configurations not shown that represent a single vertex of the triangle being the start and end of a trajectory (i.e. a static ‘trajectory’). In addition, the higher level includes a replica of the three possible target states (upper right). However, while these are considered static at the timescale of the lower level, the slower dynamics of the higher level allow this to change over time. The key distinction here is the absence of arrows between alternative target configurations at the first level. The **C**-vector represents the statistics of a prior belief that policies will lead to correct outcomes (i.e. hand and target location match). This ensures sequences of actions that lead to the realization of this goal are more plausible than those that do not. The arrows within a level indicate the allowed transitions (encoded by **B**) between these configurations. The arrows between levels show the generation of lower level variables by higher level variables. This rests upon generation of a discrete outcome via **A^(2)^**, which is then used to generate policies [via **E^(1)^**] or initial states [via **D^(1)^**]. The role of **D^(2)^**is to provide a prior belief about the initial states at the higher level. Note that, if we were to extend this model to include further levels, this would also become an empirical prior, recapitulating the role of **D^(1)^**. However, given that Level 2 is the highest level considered here, **D^(2)^**is simply a vector of prior probabilities. This says that the target states may be in any initial configuration with equal probability and that the initial state probability is equally distributed among any of the trajectories that start at the lower-right target.

Following from an assessment of reflexes, we now move to an assessment of coordination. Typically, in a clinical setting, this is done by asking a patient to reach out and touch two different objects (typically their nose and the clinician’s finger) and to alternate between the two while the clinician moves one of the targets so that it changes position each time. Based upon this idea, we constructed the generative model in Fig. 3. This comprises three levels, the lowest of which is the model in Fig. 1. The upper two levels (shown in Fig. 3) represent the processes that generate the *v* variables in Fig. 1. The arrows in this figure indicate the non-zero terms in the matrices that comprise the parameters of the generative model. The highest level of this model comprises two sorts (i.e. factors) of categorical hidden states. The first is the combined position and direction of movement of the hand (Fig. 3, top left). Consistent with dynamics described at higher levels of the cortex,^59^^,^^60^ this is a relatively coarse-grained representation spatially and temporally. The position is defined in terms of which of three spatial locations (shown as red dots) the arm starts from and the direction of movement in terms of which it is moving towards. The second factor at the highest level is where the current target is (darker sphere). This may change from moment-to-moment from the perspective of the highest level.

The lower (discrete) level of this generative model involves much more temporal and spatial detail than the higher level but covers shorter periods of time.^61^ This means that the target location is now static, as it only changes over the slower scale prescribed by the higher (discrete) level. The increased spatial resolution at the lower level affords the opportunity to represent spatial locations that are intermediate to those of the three target locations. These are shown as small red spheres. For each short trajectory at this level, the higher level specifies a starting location (via **D**) and a direction of travel (**E**) that influences the transitions (**B**). For visual clarity, we have not shown all the possible transitions between the (red) attracting points. In brief, one policy leads to a clockwise transition, one to an anticlockwise transition and one to a static transition (i.e. attracting point stays in the same location). Each of the locations at the lower level, indicated by the red spheres, maps to an attracting point (*v*_1,2,3_) in the continuous model in Fig. 1. Similarly, the shading of each of the target spheres maps to the continuous representation of target colours (*v*_4,5,6_). There is one other outcome of the categorical levels of the model that never reaches the continuous model (i.e. is conditionally independent of it). This is whether the hand position and the target position match or whether the hand has not yet reached the target. By setting a prior belief (**C**) that the correct hand position will be achieved, the course of action most likely to fulfil this attains a higher plausibility. Note that this is not used as a prior over outcomes at the level of inference about outcomes but as a prior belief about the consequences (on average) of policies. In other words, it is used as if it were a parameter of the prior over policies. The prior over policies includes both **E** and **G**, where **G** is a function of **C**. The **C**-vector may be thought of as a parameter of the prior belief over policies. However, it is an interesting quantity, as it could be thought of as a prior itself. The key idea here is that we specify a prior (**C**) over observations, that is the Bayesian model average under all policies (weighted by their respective probabilities). In place of specifying the probability of each policy and then finding this average, we specify this average and use this to express a prior over policies that would realize this (when an information theoretic bound is minimized). This is almost the opposite approach to the use of a free energy bound to approximate a marginal distribution over outcomes, as it starts from the distribution over outcomes and finds an expected free energy that defines a distribution over policies.

At this point, it is worth summarizing the way in which continuous and categorical inferences talk to one another.^16^ The key idea here is that each categorical outcome is assigned to a point in continuous space. By averaging these points based upon the relative probability of each outcome, we generate an empirical prior belief about the location of the current target in continuous space. To update beliefs about these outcomes, we can treat each outcome as an alternative hypothesis for the continuous dynamics at the low level and compute the evidence for each hypothesis. Practically, we can do this efficiently by appealing to Bayesian model reduction.^62^^,^^63^ This is a statistical technique that allows us to use the inversion of a ‘full’ model to calculate the evidence we would have achieved had we used alternative priors. The full model in this context is the continuous model where the prior expectation for the *v* variables is given by a weighted sum of all possible expectations. Each alternative (reduced) model is given by choosing an expectation associated with a specific outcome, as opposed to taking the expectation based upon all of these. Equation 4 expresses the form this takes. We use **L***_τ_*(*t*) to indicate the (log) evidence as a function of time (*t*) during a categorical epoch (*τ*). This depends upon the probability of continuous data (*y*) given categorical outcomes (*o_τ_*). This is accumulated (or integrated) and combined with the prior (**o***_τ_*) for that epoch to give a posterior belief (**r***_τ_*).

(4) $$\begin{array}{c} r_{\tau }^{\left(1\right)}=\sigma \left(\ln o_{\tau }^{\left(1\right)}+\int_{\tau }^{\tau +1}L_{\tau }(t)dt\right)\\ L_{\tau }{\left(t\right)}_{i}=\ln p(\tilde{y}(t)|o_{\tau }^{\left(1\right)}=i)-\ln p(\tilde{y}(t)|E_{Q(o_{\tau }^{\left(1\right)})}[o_{\tau }^{\left(1\right)}]) \end{array}$$

Like the negative free energies used as approximate (log) evidences elsewhere in this paper, **L** approximates a log evidence based upon an approximate posterior. The second line of Equation 4 calls upon the mappings between the discrete and continuous parts of the model that depend upon precisions formulated in terms of generalized coordinates of motion (Equation 3). This is useful to know, as it constrains the connectivity implied by the inversion (i.e. solution) of this generative model. The degree to which higher orders of motion influence the expected position of the hidden causes, and the reciprocal influence of the empirical prior precision for these higher orders, implies brain regions computing these smoothness parameters should exert an influence over those that mediate the interaction between continuous and categorical variables. In addition, note the predicted outcomes (**o***_τ_*) that appear in this equation are themselves computed by weighting the predicted outcomes under each policy by the relative probabilities of each policy (**π**). This implies both smoothness estimation and beliefs about policies should influence those regions translating between continuous and categorical inference. We will return to this crucial issue in the discussion.

**Figure 4:**
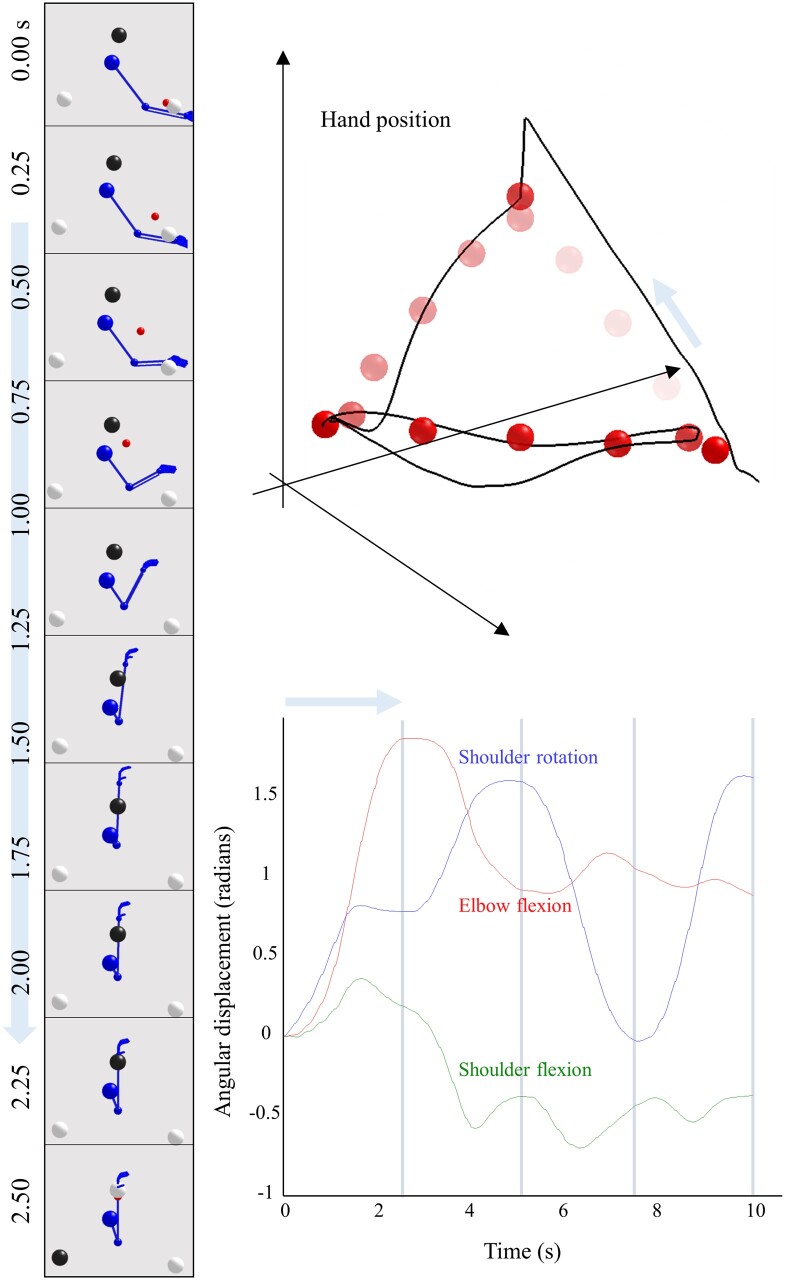
**Hierarchical movement planning and execution.** This shows healthy performance of the coordination task in Fig. 3. The upper right plot illustrates the series of attracting points inferred during the course of the movement as red spheres. These are shown as progressively darker over time (the apical sphere is darker simply because it was inferred as the target for multiple time-steps). The black line illustrates the trajectory of the hand. The blue arrow indicates the direction of travel, and the segment of the movement shown in the frames on the left. The lower right plot shows the angular coordinates for the shoulder and elbow over time. The vertical grey lines indicate the transitions at the highest level of the model, which coincide with the points at which the dark (target) sphere changes location. Note the change in shoulder flexion as the first target is reached. At the start of the trajectory, the shoulder flexes slightly. As the elbow flexes, continued shoulder flexion would bring the hand above the target. This is anticipated, and the shoulder begins to extend to prevent this from happening. Crucially, this means that the distance between the hand and the target is decreasing throughout the entire trajectory and there is no overshoot (or hypermetria). We provide an example where this fails later (Fig. 5).

Figure 4 shows what happens when we simulate inference in the model of Figs 1 and 3 by numerically integrating (i.e. solving) the equations in the Supplementary material (which solve Equation 2). This provides an example of healthy behaviour in the coordination task outlined above. The target location changes three times and, each time, the three controllable joints of the arm flex, extend or rotate such that the hand reaches the target. A series of selected frames are shown on the left of Fig. 4, which show the movement of the arm and the (imaginary) red spheres to which the arm is drawn. By predicting the proprioceptive input that would be present if there were a spring pulling the hand to these fictive targets—and by inferring a sequence of intermediate targets from the current location to the final target—spinal reflexes are engaged that resolve the discrepancy between predicted and observed proprioceptive data such that the arm reaches its target. The trajectory of the hand and the sequence of imaginary spheres to which it is drawn are shown in the top right plot in Fig. 4. The corresponding joint angles are shown in the plots below. Note that this formulation of co-ordinated motor activity dissolves Bernstein's problem,^64^ because there is one unique trajectory under the priors implicit in the generative or forward model. In other words, there are no *ad hoc* objective functions^65^ necessary to constrain the plurality of trajectories and degrees of freedom associated with any goal-directed behaviour—the only objective is to realize the movement that maximizes model evidence (or free energy). All that is required to specify this free energy functional is the set of priors that detail the structure of the task (i.e. how we as experimenters or the physical world generate the data presented to the model). Crucially, these all participate in the same objective function. Another way of putting this is that the only thing that needs specification under this approach is the problem to be solved. This includes the physics of the problem in addition to the decisions to be made and the final answer that we expect to settle upon. Once this is specified, the problem entails the objective function, which can be solved automatically and generically.

In referring to a ‘goal’, we mean there is a prior preference or expectation built into the generative model that the hand location will be congruent with the currently highlighted target (i.e. the darker sphere). To get a sense of the ‘goal’ of the arm movement, it is worth translating the generative model into the kind of instructions we might ask a participant to follow during an experiment. The highest level effectively says, ‘During this task, there are three targets that will be present throughout. One of these will be dark, and the other two light. Periodically, and randomly, we will change which of the three targets is darkened’. The lower Markov decision process level says, ‘The task here is to touch whichever is the dark target at any one time’. This is expressed by the correct and incorrect outcome that predicts ‘correct’ when the location of the hand and the dark target align and ‘incorrect’ otherwise. The **C** vector specifies that the former is preferred in this experiment.

This simulation shows how the presence of a deep temporal model naturally extends the ‘equilibrium point hypothesis’ to an ‘equilibrium trajectory hypothesis’. Importantly, this means that there are two sorts of computational syndrome we might expect when lesioning this model. The first is a failure to infer the series of attracting points leading to the goal. The second is a failure to implement the movements that lead to the realization of the kinematics implied by these attracting points. This speaks to a division into pathologies of discrete and continuous inference or, equivalently, between those affecting the planning and execution of movements. Interestingly, the same theoretical distinction between continuous and discrete processes was proposed based upon clinical observations at the end of the 19th century: ‘the cerebellum is the centre for continuous movements and the cerebrum for changing movements’.^66^

**Figure 5:**
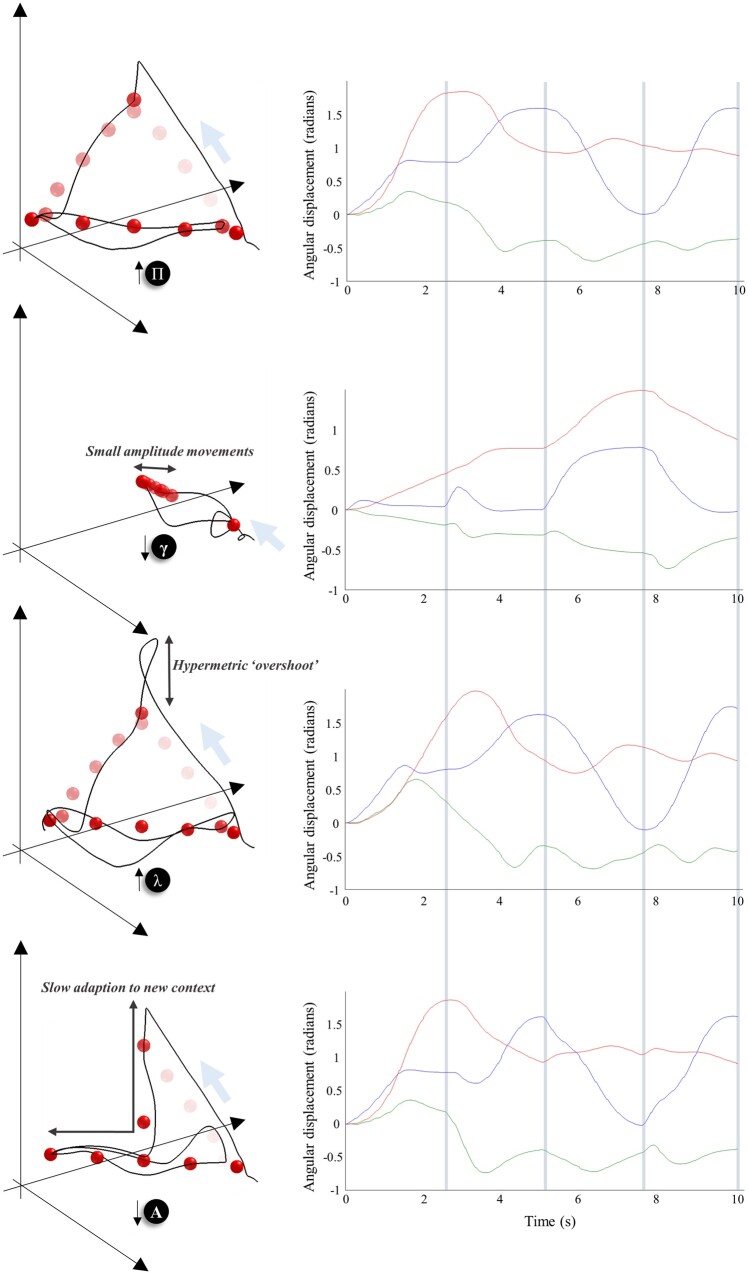
**Pyramidal and extrapyramidal.** These plots show the consequences of four specific synthetic lesions to the performance of the task shown in Fig. 4 and highlight the difference between pyramidal (i.e. corticospinal) and extrapyramidal lesions. These include the same lesions as in Fig. 2, but additionally include two perturbations to the discrete parts of the model. The first plot illustrates the preservation of coordination following overestimation of sensory precision (Π, compare with the trajectory of Fig. 4). The second illustrates a decrease in the precision of the contribution of the expected free energy to beliefs about ‘how I am going to act’ (γ). The lack of confidence in selecting a sequence of fixed points leads to their estimation as being somewhere in the middle. Note that there is no impairment in reaching these fixed points. The initialization of movement via a series of small amplitude movements resembles the ‘festinant’ gait sometimes observed in Parkinson’s disease (although this is typically observed in the lower limbs). Note the series of superimposed red spheres following this, indicating a decrease in movement amplitude following the initial movement. The third plot shows the overestimation of smoothness we saw earlier, with hypermetric overshoots at the end of each segment of the movement. The changes following overestimation of λ are strikingly similar to those plotted in Holmes^48^ for patients with cerebellar injuries. More modern studies also show the same kind of overshoot in limb trajectories.^67^ The final line shows what happens when the strength of the connections between the highest and middle levels of the hierarchy are attenuated (attenuating the precision associated with **A**). This shows successful completion of the task, but an apparent confusion each time the target changes position (often leading to a move towards the centre). While rapidly compensated for, this implies a discontinuous sequence of movements that fail to be synthesized into a coherent motor narrative.

Figure 5 illustrates the consequences of a set of model ‘lesions’ for the arm movement trajectory, where the targets follow the same sequence as in Fig. 4. Note that manipulations of the precision (Π) of sensory input do not impair the performance of this coordination task. If we interpret this lesion as in Fig. 2—interruption of descending signals from the pyramidal system—the unaffected performance is consistent with the relative preservation of coordination in patients with corticospinal lesions.^68^ More complex motor tasks, including grasping, can be associated with deficits following these lesions,^69^ but the task shown here is not sufficiently sensitive to demonstrate these. Overestimation of smoothness (λ) does not impair the planning of movement, in the sense that the same sequence of planned targets is inferred as in Fig. 4. However, there is a marked hypermetria (i.e. movement beyond the intended goal) at each target location with an oscillatory path between these locations.^70^ Recalling that no such oscillations were present at rest, before the tendon tap, in the simulations in Fig. 2, we can interpret this trajectory as expressing an intention tremor. This is characteristic of cerebellar disease and of Purkinje cell degeneration.^71^ This provides an example of disorder of movement execution, as opposed to movement planning. It also endorses the idea that the cerebellum estimates this smoothness, as we find the same lesion that induced cerebellar-like reflexes also induces the coordination deficits associated with a cerebellar syndrome.^72^^,^^73^

It is important to highlight at this point that there are many different causes of a cerebellar ataxia.^5^ These range from inherited disorders, such as the spinocerebellar ataxias,^74^ to acquired vascular,^75^ demyelinating^76^ or systemic insults.^77^ The simulations presented here speak to the common pathological end point of these processes. As such, it is worth briefly addressing theories of cerebellar pathophysiology^78^ and what this end point could be. This serves to illustrate the more general point that clinical neurology is only made possible through the fact that the number of ways in which the nervous system reacts to perturbation is much smaller than the number of pathological processes that could cause this perturbation. Understanding the mechanism of a common end point—for instance, through a computational approach as outlined here—might point to targeted therapeutics that address this mechanism, despite being agnostic to the specific aetiology. Returning to the example of cerebellar dysfunction, the parameter *λ* we deal with here directly modulates cerebellar targets, as Equation 3 suggests it contributes to the same spinal circuits targeted by the corticospinal tract. This places *λ* in the cerebellar output nuclei or red nucleus and implies any upstream damage to the cerebellum will influence this.

Physiologically, the outputs of the cerebellum are under inhibitory control from the Purkinje neurons.^79^ This makes sense of the idea that cerebellar lesions lead to overestimation of *λ*, as the neural populations encoding this variable are disinhibited. Purkinje neuron activity depends upon patterns of activity in parallel fibres that run along the surface of the cerebellar cortex and upon climbing fibres that arise from the olivocerebellar tract.^80^ Prominent theories of cerebellar function argue that climbing fibres aid in learning which patterns of parallel fibre input should prompt disinhibition of Purkinje cell targets.^81^ While there remains controversy over the synapses in which this learning takes place,^82^^,^^83^ it follows that disruption of the Purkinje cell influence over the output nuclei will also disrupt learned contextualization of this output.

These clinical and physiological observations help localize the message passing that underwrites cerebellar function and also highlight an incompleteness of the generative model. The implication is that we require explicit priors over *λ* that enable learning of this variable. As Bayesian message passing is necessarily reciprocal (if A tells us something about B, then B tells us something about A), this implies messages to the cerebellar output nuclei must be constructed from those areas influenced by *λ*—consistent with the contribution of the spinocerebellar tract to the parallel fibres.^84^

The hypothesis that the cerebellum deals in temporally correlated fluctuations makes predictions for clinical research. For example, while very important in motor control, temporal correlations are also important in other sensory systems. This implies patients with cerebellar lesions should be impaired at perceptual tasks involving motion discrimination. Motion is important here, as *λ* relates to autocorrelations over time. Evidence in favour of this includes the increased perceptual threshold required in visual dot-motion tasks in cerebellar patients compared to controls.^85^

In addition, Fig. 5 introduces two new lesions that target the discrete parts of the generative model; i.e. categorical inference or planning. The first of these is disruption of the precision associated with the contribution of expected free energy to policy selection.^86^ To understand the contribution of this, we briefly review the way in which policies are inferred under active inference. Equation 5 sets this out explicitly (in this expression, free energy and expected free energy decrease with log evidence. In statistics and machine learning, authors prefer to use the negative version of these functionals):
(5)
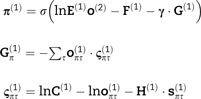


This says that posterior beliefs about policies [**π**^(1)^] comprise three parts. These are the prior plausibility of each policy [**E**^(1)^] taking account of higher level (slowly changing) contexts [**o**^(2)^] and two sorts of free energy functional [**F**^(1)^and **G**^(1)^]. These functionals score the evidence and expected evidence for a policy. The key difference between the two is that the expected log evidence (i.e. negative expected free energy) involves an expectation with respect to future (unobserved) outcomes [**o**^(1)^] as indicated by the dot product in the second line. The final line sets out the key elements of the expected free energy. The first two terms quantify the difference between predicted and preferred [**C**^(1)^] outcomes, while the third expresses the ambiguity (or conditional entropy) of state-outcome mappings expected under a policy. Technically, this corresponds to the Kullback–Leibler divergence between predicted and preferred outcomes and its minimization is known as risk sensitive or Kullback–Leibler control in optimal motor control.^87^

Together, these balance exploratory (ambiguity-averse) and exploitative (preference-driven) behaviours. Note that the contribution of the expected free energy in the first line is weighted by a precision parameter (**γ**). This quantifies the prior confidence that policies will minimize expected free energy. This precision has been repeatedly associated with dopamine signalling in both theoretical^88^^,^^89^ and empirical^90^^,^^91^ domains. It is interesting to note that this effectively weights the contribution of two opposing influences over policy selection, much as dopamine regulates the balance between the direct and indirect pathways through basal ganglia circuitry.^92^^,^^93^

Figure 5 shows that attenuating the precision parameter **γ** leads to a qualitatively different sort of behaviour than the manipulations outlined above. Here, the lack of confidence in policy selection favours a set of attracting points that are either near the start location or are in the centre of all of the other possible points. This failure to confidently predict where the hand will move to next induces either a reliance on prior beliefs about the start location, or the averaging all predictions to reach the centre. Heuristically, we can think of the first quarter of the trial as failing to accumulate enough evidence in favour of a move to a new location. The slightly larger movements in the second quarter are not enough to reach a new location but provide more substantial evidence of displacement from the start location. This facilitates a movement (in the third quarter) away from the start but to a location in the centre of all others. Finally, the drift back to the initial location could reflect the presence of the hand between the start and the centre, providing evidence in favour of a trajectory between this location and all others. In the absence of clear evidence towards another specific location, this leads to a return to the initial location. Note that there is no problem in motor execution, as the hand is drawn reliably to the red spheres. The problem is in the selection of the location of these imaginary attracting points. The difficulty in initiating large amplitude movements—and the fact that movements are slow, once initiated, are compatible with the kinds of disorders associated with subcortical lesions.^94^ These disorders include conditions like Parkinson’s disease,^95^^,^^96^ in which dopaminergic neurons of the substantia nigra pars compacta are depleted.

The final lesion shown in Fig. 5 targets the deep hierarchical structure of the discrete part of the generative model. As shown in Fig. 3, the two discrete levels are linked by the **A**^(2)^-factor. This factor is used to generate predicted outcomes, which are then mapped to prior beliefs about policies (see Equation 5) and prior beliefs about the initial state at the lower level. The reciprocal message back to the higher level then depends upon which policy and initial state were inferred by the end of the trajectory at the lower level. This is shown in Equation 6:

(6) $$\underset{\mathrm{posterior}}{\underbrace{r_{\tau }^{\left(2\right)}}}=\sigma \left(\underset{prior}{\underbrace{\ln o_{\tau }^{\left(2\right)}}}+\underset{policy\;likelihood}{\underbrace{\ln E^{\left(1\right)}\cdot \pi^{\left(1\right)}}}+\underset{state\;likelihood}{\underbrace{\ln D^{\left(1\right)}\cdot s_{1}^{\left(1\right)}}}\right)$$

The reciprocal message passing between these fast and slow levels facilitates the seamless composition of a series of trajectories into a motor narrative.

Our final lesion disrupts this message passing by attenuating the precision^97^ associated with **A**^(2)^. This effectively disconnects those neuronal populations representing where the arm is coming from and going to, from those that represent the initial position and policy at the lower level. This precludes the use of inferences about one trajectory from influencing the next. As Fig. 5 shows, this disrupts the sequence of attracting points inferred. However, it does so in a quite different way to the lesions of **γ**, which provides us with a final dichotomy: inferences about states and policies.

Functionally, this dichotomy maps onto the distinction between executive^98^ and planning^9^^,^^99^ functions; anatomically, it may be equivalent to the distinction between cortical and subcortical syndromes.^100^ One terminological conflict worth addressing is the traditional view of executive function as comprising planning, working memory and inhibition and the formal realizations of these processes. The conflict here is that all these processes (defined formally) occur over a range of timescales, while it is typically only the slower timescales that are thought of in terms of executive function. This precludes (for example) the working memory processes that mediate visual scene construction, which ensures we perceive more than the limited foveal field of vision available to us at any one time. It also means that very fast planning processes that determine the shape of a short motor trajectory are not treated as ‘executive’. Instead, executive function typically comprises working memory over the timescale of delay-period tasks^101^ and planning of the sort required to solve (for example) a Tower of Hanoi problem.^102^ Here, we use the term ‘executive’ to refer to those inferences with long temporal horizons. Operationally, in relation to this task, this means inferences about sequences of trajectories (intended to reach a certain goal state each; i.e. ‘I will first move my hand to the upper target, followed by the lower left, and then the upper target again’) qualify, but inferences about sequences of movements within a goal-directed action (i.e. ‘On my way to the upper target, I will move through these intermediate spatial locations’) do not; *cf*. motor chunking.^15^

Note that, following the synthetic lesion of **A**, each shorter trajectory (between the vertical lines) ends at the same place as in the healthy model. The points at which the trajectory deviates occur immediately after the target location changes (i.e. at each transition at the higher level of the model). This suggests a failure to change to a new sort of policy when the context changes. Despite this, the information available over a faster scale is sufficient for the appropriate trajectory to be inferred, if a little later than it would have been with an intact model.

One perspective on this—consistent with the role of the frontal cortices in coordinating working memory^103^—is that this is due to a failure of ‘immediate recall’^104^^,^^105^ of the previous part of the trajectory (before the change in target location), preventing synthesis of movement plans across changes in task context. Another perspective is that this provides an interesting connection to another well-recognized sign associated with these disorders. This is the phenomenon of perseveration—where patients who have started to engage in a given behavioural protocol struggle to abort or change this behaviour at the appropriate time.^106^^,^^107^ This lesion could underlie the perseverative or task-switching difficulties associated with frontal lobe executive syndromes^108^ with consequences in the motor domain.^109^

There is a (loose) sense in which this is the opposite of the Parkinsonian type symptoms following lesions of **γ** described above, which lead to excessive reliance on inferences about slowly changing variables at the higher level.^110^ In contrast, a disconnection or lesion to the mapping associated with **A** leads to over-reliance on inferences about fast-changing variables and a failure to deal with contextual change. The dichotomy here is one of shifting the balance to behavioural imperatives arising from slower or faster timescales. However, reducing the precision of either of these imperatives ultimately reduces the confidence in any plan of action. This means we should expect attenuated basal ganglia output and impaired task switching in both Parkinsonian and frontal syndromes—as is seen in these conditions.^108^^,^^111^ This idea lends itself to experimental evaluation, as it implies a common attenuation of basal ganglia signals in (for example) functional MRI in patients with frontal and parkinsonian syndromes compared to controls while engaging in task-switching behaviour.

## Discussion

### Computational anatomy

**Figure 6:**
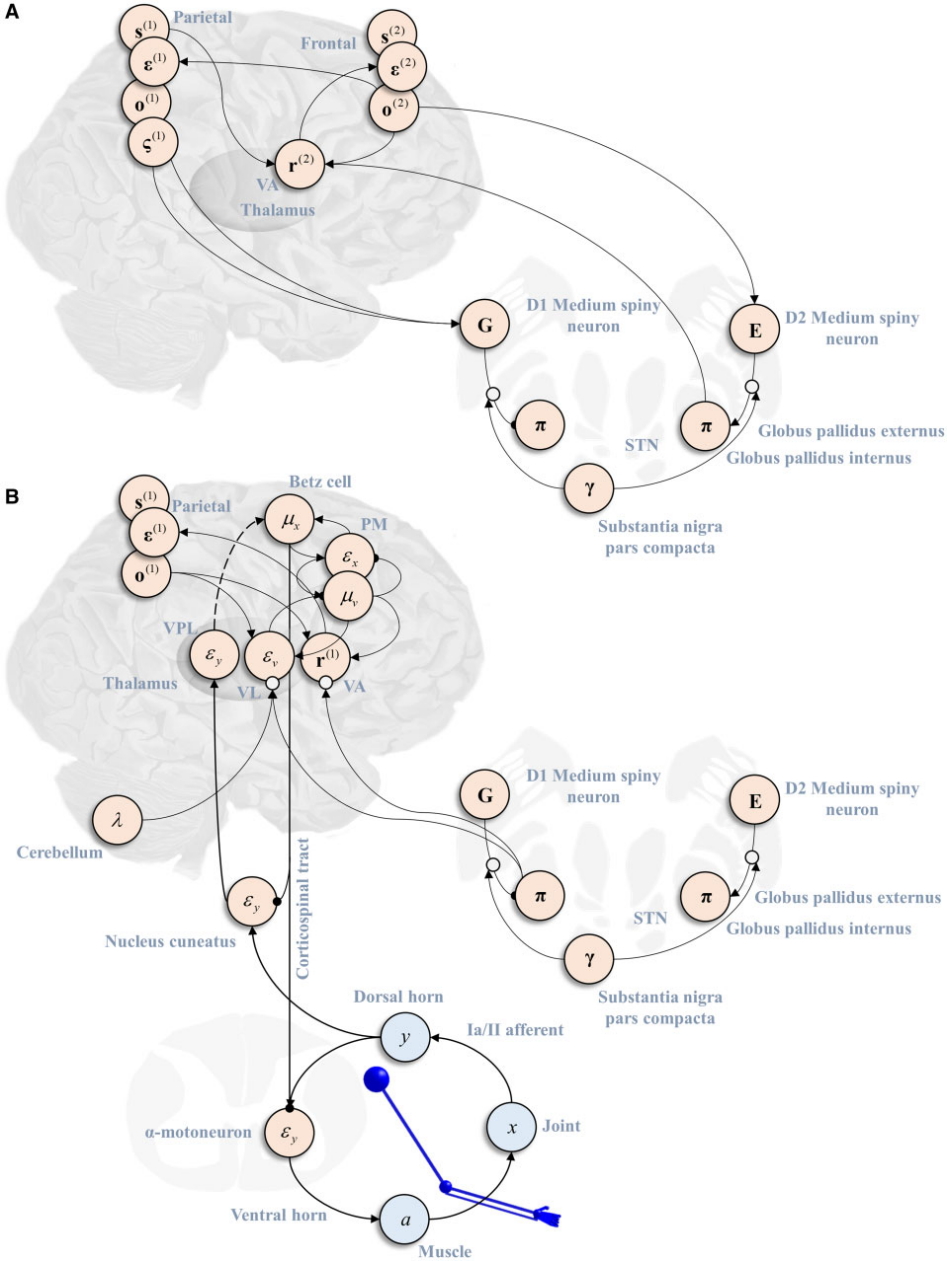
**A computational anatomy of movement.** This schematic illustrates an interpretation of the inferential message passing that underwrites the simulated movements in terms of the known anatomy of movement. (**A**) The relationship between the two levels of the discrete model. This treats estimation of hidden states as occurring in cortical columns in the frontal (slow) and parietal (fast) cortices. Each of these has an influence over planning in the basal ganglia, with the expected free energy at the lower level used to inhibit the basal ganglia output (as in the direct pathway), while the empirical priors derived from the higher level have a net excitatory effect on the output nuclei (as in the indirect pathway). Biologically, the latter is a disynaptic pathway including synapses in the globus pallidus externus and the subthalamic nuclei. The message passing shown in this schematic only deals with the net influence of this pathway. (**B**) Panel shows how the message passing of the lower level of the discrete model interacts with the continuous model. Note that the output of the basal ganglia nuclei influence the translation between hierarchical levels bi-directionally through Bayesian model averaging over policies, and in computing posterior beliefs to be passed upwards. Both the anterior and lateral parts of the ventral thalamic nuclei receive this input. The latter additionally receives cerebellar input which suggests this is the best candidate for the computation of the error at the continuous level, as this must be weighted by its associated precision, which depends upon the smoothness. We have associated the expected joint positions with the layer V Betz cells of the motor cortex. These are used to make descending predictions about proprioceptive input that are then compared to sensory afferents, leading to correction of any errors through motor neuron activation. For simplicity, we have omitted the predictions of visual data from this schematic.

In the above, we set out a generative model whose inversion or solution—based upon a form of inferential message passing with a well-defined computational anatomy—enables performance of simple motor tasks of the kind used to assess neurological function in a clinical setting. We found that synthetic lesions to the generative model resulted in motor behaviour consistent with syndrome categories observed in clinical populations (see Table 1 for a non-exhaustive summary). The mutual constraints offered by the anatomy of message passing and the consequences of lesions in relation to empirical data imply specific hypotheses about the realization of these computations in brain anatomy. Figure 6 outlines an anatomical scheme that satisfies these constraints and implicates many of the same anatomical regions as in existing schemes.^112^ At the level of the spinal cord, this shows the α-motoneurons as using the discrepancy between the proprioceptive inputs and descending predictions about these data to drive muscle contraction. Any residual error is communicated to the ventral posterior lateral thalamus via the nucleus cuneatus. This thalamic nucleus may (polysynaptically) project to the motor cortex. However, this is not strictly necessary under active inference, as the prediction error is largely suppressed through motor reflexes (Fig. 6). This idea has been used to explain the poverty of projections to cortical layer IV—the layer typically in receipt of ascending projections^113^^,^^114^—in ‘agranular’ primary motor cortex.^38^^,^^115^ This is endorsed by the increasing recognition of the central role of spinal reflexes in implementing complex coordinated behaviour.^116^

**Table 1:** Clinical and theoretical homologues

**Neurological syndrome**	**Clinical sign**	**Synthetic lesion**	**Functional role**
Cerebellar	Pendular reflexes	λ	Smoothness of random fluctuations
	Dysmetria	λ	
Corticospinal	Brisk reflexes	Π	Precision (inverse variance) of likelihood distributions
	Preserved coordination	Π	
Parkinsonian	Slow movements	**γ**	Precision (inverse temperature) of policy priors
	Small amplitude movements	**γ**	
	Delay in movement initiation	**γ**	
Executive	Impaired ‘immediate recall’	**A**	Precision (inverse temperature) of empirical (hierarchical) priors

The predictions sent from the primary motor cortex to the spinal cord themselves depend on input from other cortical regions; the most likely candidates in the context of goal-directed upper limb movement being the frontal and parietal cortices.^117–122^Figure 6 illustrates this in terms of inferences about continuous variables passed from the premotor cortex. Interactions between the premotor cortex and motor thalamus—comprising ventral lateral and ventral anterior nuclei—are shown as translating between discrete and continuous inference. Note that this translation depends upon cerebellar inputs to the ventral lateral thalamus, as this modulates the gain of its projections to the cortex, and on basal ganglia inputs to both ventral lateral and ventral anterior thalamic nuclei to take account of Bayesian model averaging over policies.^123^ This is based on the idea that the basal ganglia are engaged in evaluating alternative policies.^124^ This view of the basal ganglia is highly consistent with the view that, while alternative sequences of actions are likely represented in cortical regions, the evaluation of these alternative plans appears to take place within basal ganglia circuits.^99^^,^^125^ As we saw above, this evaluation depends upon the (expected) evidence for each policy, and hierarchically derived prior beliefs. Figure 6 depicts these influences as arising from the direct and indirect pathways from the striatum to the globus pallidus internus. Note the internal consistency of this with the role of dopamine in weighting the relative contributions of these. In addition, the direct pathway has a net inhibitory influence over the basal ganglia outputs, in virtue of the suppression of policies with high expected free energy, while the net influence of the indirect pathway is excitatory, specifying the range of plausible policies for a given context. These influences over the (GABAergic) output nuclei and their behavioural consequences are consistent with those found through optogenetic manipulation of the striatal medium spiny neurons at the origin of these basal ganglia pathways.^126^^,^^127^

### Synthetic behaviour

Notably absent from the phenomenology of the synthetic dopamine lesions is the classic parkinsonian resting tremor.^128^ This raises an important point that should contextualize the account on offer here. Syndromes like Parkinson’s disease are not consequences of focal lesions but depend upon pathogenic processes with specific anatomical distributions. This is seen in studies in which the dopaminergic midbrain is lesioned in monkeys.^129^ These also fail to induce a resting tremor (unless additional lesions are made elsewhere). In addition, aspects of the parkinsonian syndrome have been associated with dynamic dysfunction in reticular thalamocortical networks.^130^ Observations of this sort have led some to argue that a tremor is not an integral part of the syndrome induced by degeneration of the substantia nigra pars compacta.^9^ Another perspective on this is that the tremor results from a functional diaschisis,^10–12^ with other components of the motor network^131^ compromised as a consequence of the primary insult.

It is interesting to note the similarities between the synthetic trajectories under ‘cerebellar’ lesions presented here and the ‘before learning’ trajectories shown in neural network models of reaching behaviour.^132^ This raises the possibility of some mathematical equivalence between the quantities being learned in the neural network model and the optimization of precision in generalized coordinates of motion. Causal evidence in favour of extinction of learning in motor behaviour following cerebellar lesions is evident in animal research, where lesions to the subcortical nuclei^133^ or cortex^134^ both abolish previously learned motor responses and preclude their reacquisition. In addition, human studies in clinical populations have illustrated similar oscillatory motor trajectories—in a task very similar to that used here—for those with cerebellar lesions^135^ and the interaction of this with learning. In future work, we hope to unpack the optimal estimation of this precision under active inference, to see whether the implied update rules have the same form as the updates used during learning in models that seek to directly emulate cerebellar architectures. This is an important step in showing the convergence between emergent dynamics under first principles and more physiologically motivated update schemes.

While our focus has been on movement in humans, the same principles have been employed to develop synthetic and robotic systems.^30^^,^^136^^,^^137^ The reason for mentioning this is that if there are homologies between the functioning of artificial and human motor systems, both may fail in the same way. The implication is that the wealth of neurological research accumulated over the last few centuries may be vital in understanding pathologies of artificial intelligence (and vice versa).

### Hierarchical motor ‘chunking’

A key theoretical advance of our paper is the use of empirical priors about policies, as contextualized by the higher levels of a cortical hierarchy. This allows for a range of plausible action plans to be specified based upon the (slowly changing) context.^138^ This is important for three reasons. The first of these relates to the anatomy of the basal ganglia. The presence of a cortical input providing a temporally coarse influence over policy selection is highly consistent with the distribution of cortical inputs^139^ to the D2-expressing medium spiny neurons (the origin of the indirect pathway), and with their increased time-constants^140^ relative to D1-expressing neurons (of the direct pathway). It is also consistent with observations of ‘bracketing’ in the striatum,^141^ in which neural activity signals the start and end of each component (behavioural unit) of a task.^142^ Over time, repeated sequences are grouped, meaning neural activity becomes less frequent. This chunking into progressively longer action sequences is highly consistent with the idea of a hierarchy of policies (or plans) and with the idea that these are evaluated in the striatum. The second reason relates to an interesting phenomenon that arises in Parkinson’s disease. This is called kinesia paradoxa,^143^ and describes when patients who are otherwise akinetic perform fluent (often visually-guided) movements.^144^ The anatomy of Fig. 6 provides a clear hypothesis as to how this could happen. Although the direct pathway is unable to drive precise policy selection in the absence of dopamine, the indirect pathway could continue to do so, based on inferences about a slowly changing context (e.g. being on a moving bicycle) in which a particular behaviour (e.g. pedalling) is confidently predicted.^145^ The third reason for emphasizing hierarchical control of policy selection is that this provides a link between cortical and subcortical contributions to disorders in which the policies executed are incongruent with the context at hand. This is important in tic disorders (see below).

Hierarchical motor control provides an interesting connection with models that incorporate the notion of ‘effort’ in disambiguating between alternative movements.^146^^,^^147^ The key idea is that movements requiring more effort are penalized relative to less effortful choices. From an active inference perspective, effort may be equated with the implausibility of a trajectory under prior beliefs. This is sometimes phrased as a ‘complexity’ penalty, quantified by the (Kullback–Leibler) divergence between prior and posterior beliefs about how to act, or as a Bayesian Occam’s razor^148^ that tells us the simplest hypothetical action sequence tends to be the best. This suggests that the analogue of the effort penalty proposed by some is given by the prior beliefs passed from higher to lower levels via the **E**-matrix employed here. Note that, in our parkinsonian simulations, the reduced precision associated with the expected free energy means that **E** dominates inference about how to act, implying goal-directed movements driven by **G** become much more effortful. This also provides an interesting connection between neuroanatomical theories of effort^149^ which propose representation by the indirect pathway through the basal ganglia, just as with **E** in the anatomical scheme presented here.

### Alternative approaches

Some authors have argued that the forward modelling approach that underwrites the results presented here is limited in the context of faster movements.^150^ This rests partly upon the delays present in certain reflex loops, and the relationship between these and physical properties of the limbs. In contrast, others have demonstrated^151^ that this sort of modelling can be highly successful when physical parameters are closely informed by physiology. When thinking about the potential challenge fast movements might pose, it is also worth highlighting an important distinction between traditional equilibrium point theories and the implementation presented here. This is the use of generalized coordinates of motion. The advantage of representing not only the position—but also the velocity, acceleration, and subsequent orders of motion—is that higher orders of motion act as the coefficients of a local Taylor series approximation to the current trajectory. This is useful in the sense that it allows for predictions about the proximal future and past. The advantage of this is that the delays that might otherwise be difficult to deal with during fast movements can be compensated for. In addition, they provide a means of dealing with non-zero autocorrelations in fluctuations, of the sort that have been shown to have an influence over motor trajectories.^152^ For detailed numerical demonstrations of how generalized coordinates of motion can overcome the issue of neural delays during fast movements, see Perrinet *et al*.^54^

The relevance of the distinction between inverse and forward modelling for understanding disease is exemplified by the simplicity of the simulations of reflexes described above. This rests upon the idea that precision must be attenuated (by descending neurons from the cortex) under an equilibrium-point model to allow movement to take place. The hyperreflexia in Fig. 1 results from a failure of this attenuation. Inverse modelling approaches do not require this attenuation, so there is no reason to expect exaggerated reflexes following loss of descending control. More broadly, the clinical benefit of adopting the Bayesian message passing formalism advocated here is the common language it affords to address multiple interacting neurological systems. In part, this is due to the use of a single generative model to account for discrete sequences of events (i.e. planning and decision making) and continuous trajectories—which may be proprioceptive, but could also include light and sound intensity, somatosensation, or other modalities. As no brain system operates in isolation, it is important to understand the distant consequences of a focal lesion (e.g. the changes to low level motor trajectories as a consequence of prefrontal lesions shown in Fig. 4). This necessitates a common generative model of the sort on offer here.

Several computational accounts have been proposed to address specific behaviours, to provide neuroanatomical theories, or to simulate specific lesions.^153^ For example, Schweighofer *et al.*^132^ provide a model that reproduces similar cerebellar lesions to those shown here, Rigoux and Guigon,^146^ and Shadmehr *et al.*^147^ focus upon ways in which goal-directed reaching under effort constraints may be realized, and Buhrmann and Di Paolo^57^ highlight the ways in which spinal reflexes may underwrite complex motor behaviours. In this paper, we offer a single generative model whose inversion is consistent with known anatomy and qualitatively reproduces a range of clinical syndromes by lesioning the appropriate priors. This provides constraints on the functional anatomy at several different sites—and offers a way to frame multiple different sorts of lesion within the same inferential network.

It is important to note that other proposals tend to address slightly different problems to that we focus upon here. We offer a characterization of the anatomy of motor control in terms of Bayesian message passing. While there are many other computational models that could be (and have been) constructed, to our knowledge, none offer an inferential characterization of this sort. As such, these are different perspectives as opposed to competing models, per se. One point in common between this approach and those based upon inverse models is that both acknowledge the importance of an internal model of dynamics in order to engage in planning.^154^^,^^155^ The distinction is that the dynamical model and alternative plans in our approach are part of the forward model.

### Future work

One way in which we hope to exploit this formalism in future work is in understanding impulse-control disorders, such as Tourette syndrome, where there appears to be a failure to determine which policies are implausible in a given context^8^ leading to a failure to suppress involuntary movements or ‘tics’.^156^ Notably, the functional neuroanatomy of this syndrome implicates multiple regions in the cortico-subcortical loops shown in Fig. 6.^157^^,^^158^ Under the view that frontal regions normally provide the indirect pathway with contextual input, damage to either frontal or indirect pathway regions could result in the enaction of implausible (context-inappropriate) movements, through a relative facilitation of the direct pathway. This is another way of phrasing the view that the frontal cortex is engaged in contextual behavioural inhibition, of the sort investigated through ‘go no-go’ paradigms.^159^ The model provided here provides the machinery upon which such tasks could be simulated, while considering the motoric constraints on task performance. Specifically, an important issue in tic disorders is that the mechanisms generating tics and those involved in their inhibition are often difficult to disentangle.^160^ Our hope is that this form of modelling, which treats these as occurring at different temporal hierarchical levels, may be used to identify which aspects of neuronal message passing to probe empirically to disambiguate the two. The anatomical process theory associated with this message passing facilitates the expression of hypotheses answerable to, for example, neuroimaging studies. This also makes a more general point about the use of theoretical models in motivating new hypotheses to address as yet poorly understood motor pathology.

It is worth highlighting a simplification we have made in the induction of synthetic lesions. We have not distinguished between the precision afforded to different sensory modalities. In future work, we hope to exploit this to try to understand how vision and proprioception may compensate for one another. In the context of limb or hand movements, this may be useful in providing a computational characterization of dyspraxic syndromes, where lesions of the dorsal visual stream impair visually guided motor tasks.^161–163^ By selectively attenuating modality-specific precision parameters^19^ during more complex tasks (and sensory perturbations), we hope to reproduce the sorts of disconnection syndromes^164^^,^^165^ found following lesions to different parts of the brain. This affords an additional opportunity to test the construct validity of the proposed functional anatomy in relation to neuropsychological data. Furthermore, this approach could be used to model the sorts of paradigms used in healthy people to investigate multisensory integration; for example, through experimentally inducing inconsistencies between proprioceptive and visual data.^166–168^ Through an appeal to the process theories associated with active inference,^17^ synthetic neural responses may be simulated alongside behaviour, and may be used as regressors in analysis of neuroimaging data in these tasks.^91^ The hypothetical computational anatomy of Fig. 6 makes clear predictions about where each sort of neural response could be detected in different anatomical locations, and ensures the ideas presented here are answerable to empirical data.

In this paper, we have illustrated the points at which modern theoretical neurobiology has converged upon the same sorts of distinctions that are well-documented in clinical neurology. This offers a mutual validation of these approaches and takes us a step further towards a functional interpretation of anatomical systems in the brain. However, this is more than simply an intellectual exercise. Recently, we have illustrated how we can conceptualize and simulate therapeutic interventions through appealing to the same framework^89^ and illustrated how these computational parameters may be measured *in vivo*.^169^^,^^170^ Placing the consequences of pathology and therapeutic intervention in the same domain offers the potential for a functionally grounded approach to treatment development and personalized therapeutics. Ultimately, our hope is that we could estimate the parameters of these models for individual patients,^171^ based on non-invasive behavioural measurements, and simulate alternative therapeutic interventions.^172^ This would allow for highly personalized predictions about treatment responses, side-stepping a ‘trial-and-error’ approach to finding the best treatment for an individual patient.

## Conclusion

This paper has attempted to find a point of contact between modern approaches to theoretical biology and classical neurological attempts to understand the function of the nervous system. The key connection between these is evident in William Gowers’ assertion that ‘there is a region in which we must recognise hypothesis as absolute … Here we must either accept indirect perception, or we must be content with no perception of the causes of that which we observe. Where we have no certainty we must be content with probability’.^173^ This statement, while originally intended from the perspective of a neurologist, is equally applicable from the perspective of a nervous system, and emphasizes the importance of a generative model of the causes of sensory input (observations). We have illustrated how specifying a minimal generative model for movement entails an inferential architecture that is highly consistent with known neuroanatomy. The sorts of pathology this lends itself to are consistent with the classification of syndromes in clinical practice, which constitutes an important step in bridging clinical and theoretical approaches to neurobiology.

### Data availability

Data sharing not applicable to this article as no datasets were generated or analysed during the current study.

### Code availability

Although the generative model changes from application to application, the belief updates described in this paper are generic and can be implemented using standard routines (here spm_MDP_VB_X.m). These routines are available as MATLAB code in the SPM academic software: http://www.fil.ion.ucl.ac.uk/spm/. The simulations reported above can be reproduced (and customized) via a graphical user interface by inputting ‘≫ DEM’ and selecting the ‘Movement planning’ demo.

## Funding

T.P. is supported by the Rosetrees Trust (Award Number 173346). J.L. is supported by the European Union’s Horizon 2020 programme (Marie Skłodowska-Curie grant No 749988). K.F. is a Wellcome Principal Research Fellow (Ref: 088130/Z/09/Z). The Centre for Tactile Internet with Human-in-the-Loop is funded by the German Research Foundation (DFG, Deutsche Forschungsgemeinschaft) as part of Germany’s Excellence Strategy—EXC 2050/1—Project ID 390696704.

## Competing interests

The authors report no competing interests.

## Supplementary material

Supplementary material is available at *Brain* online.

## Supplementary Material

awab085_Supplementary_Data
