# Supplementary material for: The computational neurology of movement under active inference
Source: Brain. 2021 Mar 11;144(6):1799–818. doi: 10.1093/brain/awab085 (PMC8320263; doi:10.1093/brain/awab085)
Supplement: awab085_Supplementary_Data [file awab085_supplementary_data.zip › awab085-suppl_data/brain-2020-00565-File010.pdf]

The computational neurology of movement under active inference

Thomas Parr[1*], Jakub Limanowski[1], Vishal Rawji[2], Karl Friston[1]

[1] Wellcome Centre for Human Neuroimaging, Queen Square Institute of Neurology, University College London, WC1N 3BG, UK.

[2] Department of Neuromuscular Diseases, Queen Square Institute of Neurology, University College London, WC1N 3BG, UK.

*thomas.parr.12@ucl.ac.uk

Supplementary appendix 1 – Message passing and active inference

This appendix provides a technical outline of the equations shown in Figure S1.1 that solve Equation 2 in the main text for the model outlined in Figures 1 and 3. The architecture of the requisite message passing forms the basis for the computational anatomy set out in Figure 6. This appendix unpacks the message passing within a single level of a discrete generative model with and without policies, the extension of this to a hierarchical model, and the continuous message passing required to translate this into dynamics at the level of sensory input.

Marginal message passing for Hidden Markov Models

Our decomposition of Figure S1.1 begins with the general form of a Hidden Markov Model (HMM), which appears as part of the Markov Decision Process (MDP) at the first discrete level of the model, and on its own at the second, highest, level of the model. In this subsection, we unpack the marginal message passing scheme (Parr *et al.*, 2019) used to solve HMMs. These models describe how states (s) evolve over a sequence of discrete time-steps. This evolution starts with a prior probability given by a vector (**D**) of probabilities. In the blue panel of Figure S1.1, this is shown as a matrix that allows for alternative probability distributions (columns of **D**) under different observations generated by a higher level of the model. The transition from a state at one time-point to the next is given by a matrix of transition probabilities (**B**), which differs for alternative policies (π). In this sense, each policy can be seen as representing an alternative HMM. At each time-point, states give rise to outcomes (o) as determined by a matrix of likelihood probabilities (**A**).

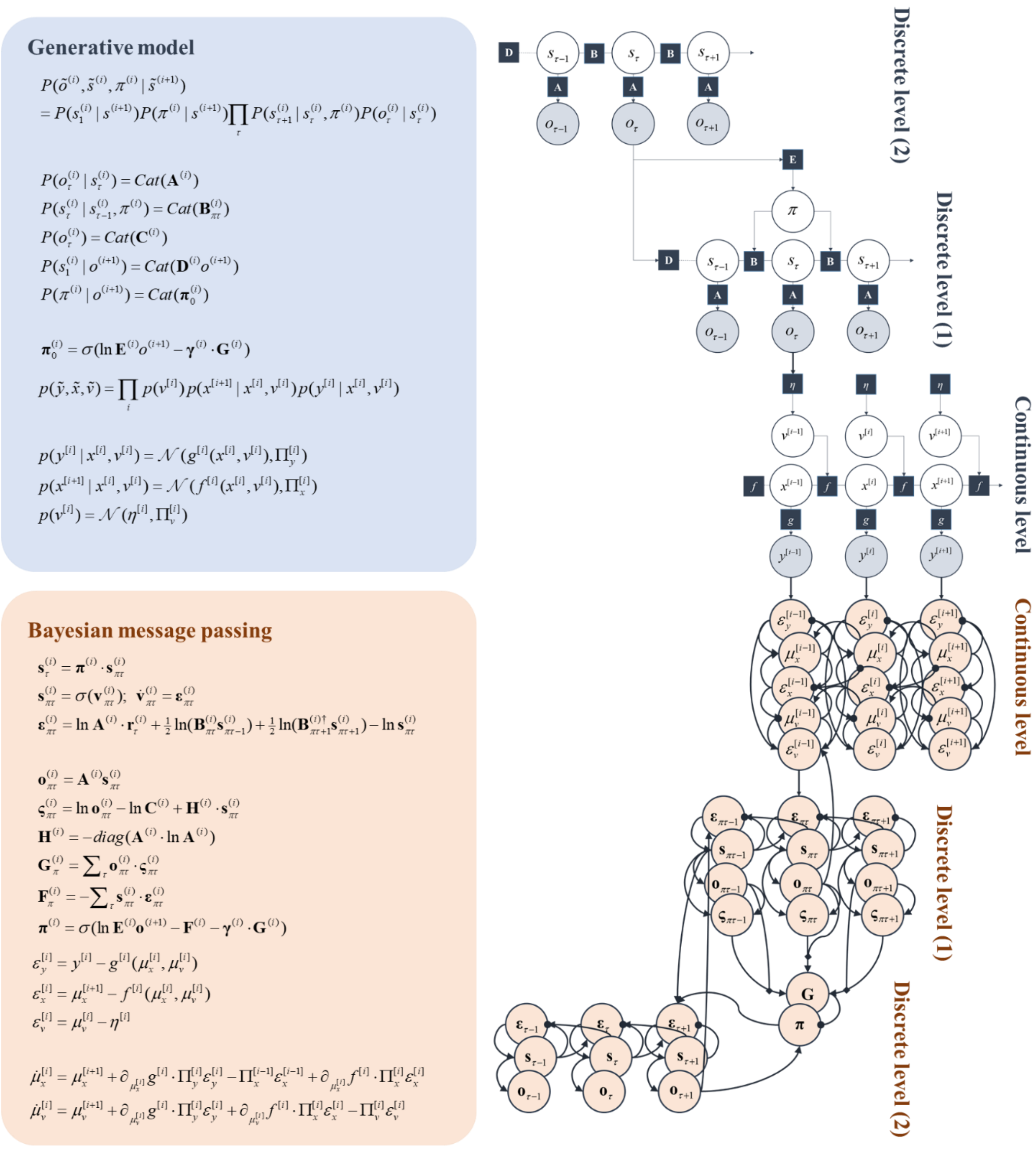

Figure S1.1 – Generative (forward) models and message passing.

The upper (blue) part of this schematic illustrates the structure of the generative model used in this paper; specified in the equations in the blue panel, and graphically as a factor graph (Loeliger *et al.*, 2007) on the right. The factor graph shows how variables (in circles) depend upon one another via probability distributions (in squares). An arrow from one circle to another, via a square, means that the second variable depends upon the first. The model is 'mixed' in that it comprises two discrete levels and one continuous level. Each discrete level describes a succession of hidden states (s) through time with dynamics determined by the **B**-factors. These generate outcomes (o), as prescribed by the **A**-factors. The outcomes generated at the highest level themselves generate short trajectories at the next level down. They do so by influencing the initial hidden state at the lower level (via the **D**-factor) and the policy (π) or sequence of transitions (via the **E**-factor). The outcomes at this level generate short trajectories at the continuous level below, by specifying the mean (η) of a normally distributed variable (v) that mediates the interaction

between the position ($x^{[0]}$), velocity ($x^{[1]}$), and higher order temporal derivatives of continuous hidden states. These generate continuous variables (y). The lower (pink) part of the figure shows the message passing that may be used to draw inferences about the variables in the generative model. The **r** variable that appears in the update equations on the left represents a posterior belief about the alternative models at the level below. For now, this can be thought of as data, from the perspective of the level at which it appears. We unpack the corresponding calculation in the main text, as it varies depending upon whether we translate between continuous and discrete, or discrete and discrete, levels. These equations can be obtained by defining a variational bound on the evidence for the model in blue and taking the derivatives of this bound with respect to the sufficient statistics of (Bayesian) beliefs about the variables in the model. For details, please see (Friston *et al.*, 2017b; Parr and Friston, 2018). Note the symmetry between the model and the message passing that inverts it. This emphasises that, while the variational inference scheme is generic, the form of the generative model is crucial in elaborating the inferential anatomy required for any given application. For the message passing scheme, we use arrows to represent excitation and circles to indicate inhibition.

Variational inference optimises an evidence bound (Dayan *et al.*, 1995; Beal, 2003) which, from the perspective of the HMM is the probability of outcomes given the specific HMM (i.e., the policy it represents). There are many ways of solving this inference problem, including belief propagation or variational message passing. In this paper, we employ a method known as marginal message passing which approximates the performance of the former while exploiting the simplicity of the latter. Marginal message passing optimises the evidence at a given time-step, conditioned upon the outcomes at other times:

$$\begin{aligned}
\ln P(\tilde{o} \mid \pi) &= \ln P(o_\tau \mid \pi, \tilde{o}_{\backslash\tau}) + \ln P(\tilde{o}_{\backslash\tau} \mid \pi) \\
\ln P(o_\tau \mid \pi, \tilde{o}_{\backslash\tau}) &= E_{P(s_\tau \mid \pi, \tilde{o})}[\ln P(s_\tau \mid \pi, \tilde{o}_{\backslash\tau}) + \ln P(o_\tau \mid s_\tau) - \ln P(s_\tau \mid \pi, \tilde{o})] \\
&\geq E_{Q(s_\tau \mid \pi)}[\underbrace{\ln P(s_\tau \mid \pi, \tilde{o}_{\backslash\tau})}_{prior} + \underbrace{\ln P(o_\tau \mid s_\tau)}_{likelihood} - \underbrace{\ln Q(s_\tau \mid \pi)}_{approx.posterior}] \\
Q(s_\tau \mid \pi) &\approx P(s_\tau \mid \pi, \tilde{o})
\end{aligned} \qquad \text{(S1.1}$$

The inequality in the third line expresses an approximation to the log evidence associated with time τ. This approximation is known as a negative free energy. As written here, this rests upon use of a prior that depends upon all outcomes at other times. To compute this, we note that the marginal of two pairwise factors of the generative model gives a squared version of the quantity we are interested in:

$$\sum_{s_{\tau-1}} \sum_{s_{\tau+1}} P(s_\tau, s_{\tau-1} \mid \pi, \tilde{o}_{\backslash\tau}) P(s_\tau, s_{\tau+1} \mid \pi, \tilde{o}_{\backslash\tau}) = P(s_\tau \mid \pi, \tilde{o}_{\backslash\tau})^2 \qquad \text{(S1.2}$$

Conceptually, this is like the construction of a distribution under a Bethe assumption (Yedidia *et al.*, 2005), which uses a product of pairwise factors. The Bethe approach divides both sides by the singleton marginal density to ensure that summing over all other variables gives the (non-squared) marginal.

Here, we instead take the square root of this, and use the approximate posteriors from other time steps to find a tractable expression for the prior:

$$
\begin{aligned}
P(s_\tau \mid \pi, \tilde{o}_{\backslash\tau}) &= \left(\sum_{s_{\tau-1}} \sum_{s_{\tau+1}} P(s_\tau, s_{\tau-1} \mid \pi, \tilde{o}_{\backslash\tau}) P(s_\tau, s_{\tau+1} \mid \pi, \tilde{o}_{\backslash\tau})\right)^{\frac{1}{2}} \\
&= \left(\sum_{s_{\tau-1}} P(s_\tau, s_{\tau-1} \mid \pi, \tilde{o}_{\backslash\tau})\right)^{\frac{1}{2}} \left(\sum_{s_{\tau+1}} P(s_\tau, s_{\tau+1} \mid \pi, \tilde{o}_{\backslash\tau})\right)^{\frac{1}{2}} \\
&\approx E_{Q(s_{\tau-1}|\pi)}[P(s_\tau \mid s_{\tau-1}, \pi)]^{\frac{1}{2}} E_{Q(s_{\tau+1}|\pi)}[P(s_\tau \mid s_{\tau+1}, \pi)]^{\frac{1}{2}}
\end{aligned}
\quad \text{(S1.3}
$$

Substituting this approximate prior into the third line of S1.1, we reach an expression for the local 'marginal' free energy associated with the time-step of interest:

$$
\begin{aligned}
\mathbf{F}_{\pi\tau} = E_{Q(s_\tau|\pi)}[&\underbrace{\ln Q(s_\tau \mid \pi)}_{\textit{approx. posterior}} \\
&\underbrace{-\tfrac{1}{2}(\ln E_{Q(s_{\tau-1}|\pi)}[P(s_\tau \mid s_{\tau-1}, \pi)] + \ln E_{Q(s_{\tau+1}|\pi)}[P(s_\tau \mid s_{\tau+1}, \pi)])}_{\textit{approx. prior}} \\
&- \underbrace{\ln P(o_\tau \mid s_\tau)}_{\textit{likelihood}}]
\end{aligned}
\quad \text{(S1.4}
$$

Now that we have an expression for the free energy associated with a given marginal posterior, we can construct a gradient descent on this quantity by plugging in the sufficient statistics of the relevant probability distributions. These are simply the vectors and matrices shown on the left of Figure S1.1 and the statistics of the approximate posterior (**s**), which take the form of a vector of probabilities. Taking the derivative of this, we can construct a gradient descent procedure that minimises this free energy.

$$
\begin{aligned}
\mathbf{F}_{\pi\tau} &= \mathbf{s}_{\pi\tau} \cdot (\ln \mathbf{s}_{\pi\tau} - \tfrac{1}{2}(\ln(\mathbf{B}_{\pi\tau}\mathbf{s}_{\pi\tau-1}) + \ln(\mathbf{B}^{\dagger}_{\pi\tau+1}\mathbf{s}_{\pi\tau+1})) - \ln \mathbf{A} \cdot \mathbf{r}_\tau) \\
\boldsymbol{\varepsilon}_{\pi\tau} &= -\nabla_{\mathbf{s}_{\pi\tau}} \mathbf{F}_{\pi\tau} \\
\dot{\mathbf{v}}_{\pi\tau} &= \boldsymbol{\varepsilon}_{\pi\tau} \\
\mathbf{s}_{\pi\tau} &= \sigma(\mathbf{v}_{\pi\tau})
\end{aligned}
\quad \text{(S1.5}
$$

In Equation S1.5, the dagger symbol (†) indicates the reversed transition probability. If we started with a matrix of joint probabilities for the current and previous time, **B** is the matrix we get on normalising the columns (i.e., probability of current given previous state) while $\mathbf{B}^{\dagger}$ is the matrix obtained by normalising the rows and transposing (i.e., probability of previous state given current state). At $\tau = 1$, **B** = **D**. The variable **r** represents the vector of probabilities for outcomes. The variables **v** and **ε** are

auxiliary variables that play the role of depolarisations and prediction errors, respectively. The approximate posterior is obtained from the former via a softmax (normalised exponential) function (σ).

Markov decision processes and policies

An MDP is a set of alternative HMMs (i.e., alternative policies) that differ in their **B-**matrices, equipped with a prior over the relative plausibility of each. The prior used here is that the most probable policies are those that minimise their expected free energy (Friston *et al.*, 2017a). The expected free energy has the form:

$$\begin{aligned} \mathbf{G}_{\pi\tau} &= -\mathbf{o}_{\pi\tau} \cdot \varsigma_{\pi\tau} \\ \varsigma_{\pi\tau} &= \ln \mathbf{C} - \ln \mathbf{o}_{\pi\tau} - \mathbf{H} \cdot \mathbf{s}_{\pi\tau} \\ \mathbf{o}_{\pi\tau} &= \mathbf{A}\mathbf{s}_{\pi\tau} \\ \mathbf{H} &= -diag(\mathbf{A} \cdot \ln \mathbf{A}) \end{aligned} \quad \text{(S1.6}$$

This uses an auxiliary variable ς, which plays a similar role to the **ε** term in Equation S1.5 but is in the space of predicted outcomes as opposed to states. This is computed by taking the difference between preferred outcomes (**C**) and predicted outcomes under a policy (**o**) and adjusting this based upon the expected conditional entropy (**H**) of the likelihood (**A**). The interesting thing about the form of the expected free energy is that it behaves a little like an inverted form of the free energy. In place of comparing priors and posteriors for states, it does so for outcomes. In place of the dot product between a likelihood and outcome (as in Equation S1.5), there is an analogous dot product using the entropy and states. One way of thinking about this is that the expected free energy scores the evidence as if state trajectories were being generated by anticipated (preferred) outcomes.

To construct a prior based upon this, we use a first order polynomial in terms of **G** in log space to give:

$$\begin{aligned} \ln \boldsymbol{\pi}_0 &= \ln \mathbf{E} - \boldsymbol{\gamma} \cdot \mathbf{G} \\ &\Rightarrow \boldsymbol{\pi}_0 = \sigma(\ln \mathbf{E} - \boldsymbol{\gamma} \cdot \mathbf{G}) \\ P(\pi) &= Cat(\boldsymbol{\pi}_0) \end{aligned} \quad \text{(S1.7}$$

The constant **E** vector here provides a bias term that we will use later to enable higher levels to contextualise policy choices. To find the posterior probability for a given policy, we construct a lower bound on the evidence as above and find its maximum:

$$\begin{aligned}\ln P(o_\tau \mid \pi) &\geq E_{Q(\pi)}[\ln P(\pi, o_\tau) - \ln Q(\pi)] \\ &= E_{Q(\pi)}[\underbrace{\ln P(\pi)}_{=\ln \mathbf{E}-\gamma\cdot\mathbf{G}} + \underbrace{\ln P(o_\tau \mid \pi)}_{=-\mathbf{F}} - \underbrace{\ln Q(\pi)}_{=\ln \boldsymbol{\pi}}] \\ &= \boldsymbol{\pi}\cdot(\ln \mathbf{E} - \boldsymbol{\gamma}\cdot\mathbf{G} - \mathbf{F} - \ln \boldsymbol{\pi})\end{aligned} \qquad \text{(S1.8}$$

$$\sigma(\ln \mathbf{E} - \boldsymbol{\gamma}\cdot\mathbf{G} - \mathbf{F}) = \arg\max_{\boldsymbol{\pi}}\left\{\boldsymbol{\pi}\cdot(\ln \mathbf{E} - \boldsymbol{\gamma}\cdot\mathbf{G} - \mathbf{F} - \ln \boldsymbol{\pi})\right\}$$

When dealing with the evidence accumulated over multiple time-steps, we simply accumulate the log probabilities (i.e., **G** and **F**) over time.

Hierarchical inference

The key moves to go from the equations above to a hierarchical scheme are to change **D** and **E** from vectors to matrices (Friston *et al.*, 2017c). Each column then represents the priors for a given level conditioned upon an outcome at the higher level. This has two consequences. The first is that these priors get into the estimation of posterior beliefs for policies and (initial) states by altering the priors as follows:

$$\begin{aligned}P(\pi^{(i)} \mid \mathbf{s}^{(i+1)}) &= Cat(\boldsymbol{\pi}_0^{(i)}) \\ \boldsymbol{\pi}_0^{(i)} &= \sigma(\ln \mathbf{E}^{(i)} \underbrace{\mathbf{A}^{(i+1)}\mathbf{s}^{(i+1)}}_{\mathbf{o}^{(i+1)}} - \boldsymbol{\gamma}^{(i)}\cdot\mathbf{G}^{(i)}) \\ P(s_1^{(i)} \mid \mathbf{s}^{(i+1)}) &= Cat(\mathbf{D}^{(i)} \underbrace{\mathbf{A}^{(i+1)}\mathbf{s}^{(i+1)}}_{\mathbf{o}^{(i+1)}})\end{aligned} \qquad \text{(S1.8}$$

The second consequence is that we can treat the posterior predictive beliefs about outcomes (**o**) from the higher level as if they were priors from the perspective of the lower level, and can use the policies and initial states at the lower level to form ‘posterior’ beliefs about these (**r**) to pass back to the higher level:

$$\begin{aligned}\underbrace{\mathbf{r}^{(i+1)}}_{posterior} &= \sigma(\underbrace{\ln \mathbf{o}^{(i+1)}}_{prior} + \underbrace{\ln \mathbf{E}^{(i)}\cdot\boldsymbol{\pi}^{(i)}}_{policy\ likelihood} + \underbrace{\ln \mathbf{D}^{(i)}\cdot\mathbf{s}_1^{(i)}}_{state\ likelihood}) \\ \mathbf{o}^{(i)} &= \underbrace{\boldsymbol{\pi}^{(i)}\cdot\mathbf{A}^{(i)}\mathbf{s}_\pi^{(i)}}_{BMA}\end{aligned} \qquad \text{(S1.9}$$

The first line here treats policies and states as if they were data modalities (and **E** and **D** as the likelihood matrices that generate these from higher level outcomes). The second line shows the Bayesian model average (BMA) over policies used to compute the empirical prior for observations. Note that Equation

S1.9 assumes an interface between two discrete models and would not be appropriate for the interface between a discrete and a continuous model.

Active Bayesian filtering

The continuous level of the generative model deals with latent variables (x and v) that are represented in generalised coordinates of motion (see Supplementary appendix 3). This is a method for representing short trajectories based upon the coefficients of a Taylor series expansion, as opposed to the sequential representations used in the discrete time model above (Friston *et al.*, 2010). The key to specifying these kinds of models is to write down a pair of equations that generate data from latent variables, and that set out the dynamics of those variables:

$$\begin{aligned} \dot{x} &= f(x,v) + \omega_x \\ y &= g(x,v) + \omega_y \end{aligned} \tag{S2.1}$$

By assuming a normal distribution for the fluctuations (ω), centred on zero and with precision Π, this gives us a series of conditional probabilities that comprise the generative model ($p(\tilde{y}, \tilde{x}, \tilde{v})$). As in the discrete models above, we then construct a free energy bound on model evidence and optimise this. This bound takes the form:

$$\begin{aligned} F[\tilde{\mu}, \tilde{y}] &= E_q[\ln q(\tilde{x},\tilde{v}) - \ln p(\tilde{y},\tilde{x},\tilde{v})] \\ &\approx \underbrace{-\tfrac{1}{2}\ln 2\pi\left|C\right|}_{E_q[\ln q(\tilde{x},\tilde{v})]} + \underbrace{\tfrac{1}{2}\tilde{\varepsilon}\cdot\tilde{\Pi}\tilde{\varepsilon} + \tfrac{1}{2}\ln 2\pi\left|\tilde{\Pi}\right|}_{-E_q[\ln p(\tilde{y},\tilde{\mu})]} \underbrace{-\tfrac{1}{2}tr(C\partial_{\tilde{\mu}}^2 \ln p(\tilde{y},\tilde{\mu}))}_{-\frac{1}{2}E_q[\Delta\tilde{\mu}\cdot\partial_{\tilde{\mu}}^2 \ln p(\tilde{y},\tilde{\mu})\Delta\tilde{\mu}]} \\ \tilde{\varepsilon} &\triangleq \begin{bmatrix} \tilde{\varepsilon}_y \\ \tilde{\varepsilon}_x \\ \tilde{\varepsilon}_v \end{bmatrix} = \begin{bmatrix} \tilde{y} - \tilde{g}(\tilde{\mu}_x, \tilde{\mu}_v) \\ D\tilde{\mu}_x - \tilde{f}(\tilde{\mu}_x, \tilde{\mu}_v) \\ \tilde{\mu}_v - \tilde{\eta} \end{bmatrix} \\ \tilde{\Pi} &\triangleq \begin{bmatrix} \tilde{\Pi}_y & & \\ & \tilde{\Pi}_x & \\ & & \tilde{\Pi}_v \end{bmatrix} \end{aligned} \tag{S2.2}$$

Here, D is a derivative operator that shifts the elements of a vector in generalised coordinates of motion up by one. The second line assumes a Gaussian form for the variational posterior density[1] with mode μ and covariance C. The first term is simply the entropy of a Gaussian distribution. The second, third, and fourth terms come from (the expectation of) a second order Taylor series expansion of the log joint

[1] $p(x,v \mid y) \approx q(x,v) = \mathcal{N}(\mu, C^{-1})$

probability around the mode of the variational posterior[2]. The form of the free energy here has a useful consequence. Taking the derivative with respect to the posterior covariance, we find that the covariance that minimises the free energy can be expressed in terms of the posterior mean:

$$\begin{aligned} \partial_C F &= -\tfrac{1}{2}C^{-1} - \tfrac{1}{2}\partial_{\tilde{\mu}} \ln p(\tilde{y}, \tilde{\mu}) = 0 \Leftrightarrow \\ C^{-1} &= -\partial_{\tilde{\mu}}^2 \ln p(\tilde{y}, \tilde{\mu}) \end{aligned} \qquad \text{(S2.3}$$

Substituting this into Equation S2.2 turns the final term into a constant. In addition (due to the quadratic approximation) the first and third terms (representing curvatures) are constant with respect to the expectation. We can now express inference in terms of a gradient descent on the free energy with respect to the posterior mean:

$$\begin{aligned} &\dot{\tilde{\mu}} = D\tilde{\mu} - \nabla_{\tilde{\mu}}\tilde{\varepsilon} \cdot \tilde{\Pi}\tilde{\varepsilon} \\ &\Rightarrow \\ &\dot{\tilde{\mu}}_x - D\tilde{\mu}_x = \nabla_{\tilde{\mu}_x}\tilde{g} \cdot \tilde{\Pi}_y\tilde{\varepsilon}_y - D \cdot \tilde{\Pi}_x\tilde{\varepsilon}_x + \nabla_{\tilde{\mu}_x}\tilde{f} \cdot \tilde{\Pi}_x\tilde{\varepsilon}_x \\ &\dot{\tilde{\mu}}_v - D\tilde{\mu}_v = \nabla_{\tilde{\mu}_v}\tilde{g} \cdot \tilde{\Pi}_y\tilde{\varepsilon}_y + \nabla_{\tilde{\mu}_v}\tilde{f} \cdot \tilde{\Pi}_x\tilde{\varepsilon}_x - \tilde{\Pi}_v\tilde{\varepsilon}_v \end{aligned} \qquad \text{(S2.4}$$

Note that this gradient descent is expressed such that when the gradient is zero, the rate of change of the posterior mean is equal to its temporal derivative, not to zero. This is the role of the $D\tilde{\mu}$ term. Under active inference, we also need to minimise the free energy through action. As the only variable in the above that depends upon action is y, we can express action as:

$$\dot{a} = -\nabla_a \tilde{y}(a) \cdot \tilde{\Pi}_y\tilde{\varepsilon}_y \qquad \text{(S2.5}$$

The involvement of action only at the level of sensory input is highly consistent with the notion of a reflex arc (Adams *et al.*, 2013), of the sort found in the brainstem and spinal cord, where efferent motor neurons project to muscles and correct any deviation between incoming proprioceptive data and descending predictions about these data. These equations have been used extensively to model a wide range of neurobiological phenomena, including attention (Feldman and Friston, 2010), perceptual illusions (Brown and Friston, 2012), action-observation (Friston *et al.*, 2011), communication (Friston and Frith, 2015), and motor control (Perrinet *et al.*, 2014; Baltieri and Buckley, 2019).

To connect this to the discrete level, all we need to do is to associate every alternative discrete outcome with a different value for the prior at the continuous level. For simplicity, we assume the precision does not change, so instead only change the expectation (η). If $\boldsymbol{\eta}$ is an array of possible values of η for each

[2] The linear term of the expansion vanishes under expectation, as the expected difference between a random variable and its mode is zero under Gaussian assumptions.

outcome, the message passed from the discrete to the continuous level is this weighted by the relative probability of each outcome:

$$\begin{aligned}
&\eta = \boldsymbol{\eta} \cdot \mathbf{o}^{(1)} \\
&p(v \mid o^{(1)}) = \mathcal{N}(\boldsymbol{\eta}_{o_\tau}, \Pi_v) \\
&p(v \mid \mathbf{o}^{(1)}) = \mathcal{N}(\eta, \Pi_v)
\end{aligned} \tag{S2.6}$$

The message passed back to the higher level is based upon the accumulated evidence for each alternative outcome. Practically, this uses Bayesian model reduction to compare the evidence for a full model (third line of Equation S2.6) with a series of alternative models (second line of Equation S2.6).

Supplementary appendix 2 – Equilibrium points, inverse models, and minimum intervention

The approach we have adopted is closely in line with the equilibrium point hypothesis. However, this is one of two broad approaches that are commonly pursued in the motor control field. This appendix seeks to situate our approach relative to other computational accounts of movement, to highlight the points of contact and divergence, and to provide a degree of construct validity through reproducing one of the key results of the inverse modelling approach; the so called minimum intervention principle (McNamee and Wolpert, 2019).

Comparison with inverse modelling

The two dominant perspectives on motor control rely upon forward models that predict the consequences of actions. The point of contention is whether it is necessary to additionally employ an inverse model that predicts the best motor command given a goal, or whether we can do without this additional model (Friston, 2011). The former is the approach pursued in ‘optimal motor control’ perspectives (Egger *et al.*, 2019; McNamee and Wolpert, 2019). The latter instead relies upon the view that the proprioceptive predictions under the forward model drive movement and therefore can be treated as motor ‘commands’ (Feldman and Levin, 2009; Adams *et al.*, 2013). It has been argued that the second view accounts for neurobiological findings, including the relative sparsity of granule cells in the primary motor cortex (Shipp *et al.*, 2013). The argument here goes that, if predictions propagated from the motor cortex to the spinal cord are fulfilled (i.e., enacted by muscle movement) at the spinal level, the resolution of any prediction errors at this level makes messages passed back to the motor cortex almost entirely redundant. As granule cells are those in receipt of ascending input from sensory areas (Bastos *et al.*, 2012), there is relatively little need for them in an area whose ascending inputs should be completely determined by the descending messages it gives rise to. Note that one scenario in which ascending messages become more important here is when the movement cannot be completed due to some form of mechanical obstruction. This suggests that there are some circumstances that require spinal to motor cortical connectivity. We simulate one such example in the next section.

An additional interesting distinction between these approaches relates to the role of sensory attenuation. The key question here is why self-generated sensory input is not experienced in the same way as sensations generated by external factors. Under inverse modelling approaches, the argument goes that self-generated sensory input may be cancelled out by the predictions of the forward model under a given motor command (Blakemore *et al.*, 2000). The active inference approach deviates from this, as there is no distinction between the motor command and forward model. Instead, the explanation given here is that, in order to produce movement (through prediction), it is necessary to suppress signals that contradict this prediction to preclude its revision. This is important at the initiation of a movement. Ignoring current proprioceptive input in order to initiate a movement implies turning down its gain (i.e., a multiplicative as opposed to additive effect). In other words, the motor cortex must predict not only the proprioceptive consequences of a movement, but also that the precision of sensory input should be attenuated at the start of the movement (Brown *et al.*, 2013). This is of particular relevance for this paper, given the view that corticospinal lesions can be reproduced by a failure of attenuation of precision. As highlighted in the main text, disrupting those descending corticospinal influences that would normally attenuate precision implies a multiplicative increase in the influence of proprioceptive input. This is used to account for the brisk tendon reflexes observed following upper motor neuron lesions. The view that predicted sensory signals are simply cancelled out does not appear to account for this exaggerated response to sensory stimuli.

While these approaches inherit from different theoretical positions, they are not without their overlaps. An interesting point of connection between these approaches is the role of inverse models and expected free energies. As we see based upon the discussion under Equation S1.6 above, the expected free energy could be thought of as the free energy under an expected inverse model, in the sense that it has the form of a free energy that behaved as if outcomes were generating state trajectories. By specifying desired (anticipated) outcomes, this then finds the policies that are most likely to realise the state trajectories that lead to these desired outcome distributions. There are two important differences here. The first is that the expected free energy is absorbed into the forward model as a prior. The second is that the active inference approach to this uses current *beliefs* about states of the world, as estimated based upon the forward model, to select between plausible policies such as to bring about outcomes consistent with a preferred *distribution* over outcomes. The distinction between belief-based and goal-based scoring of alternative policies may provide the simplest method for disambiguating between the two approaches behaviourally. The next section of this appendix unpacks this further.

While the approach we have pursued here is framed as an attempt to understand the anatomy of motor control in terms of inferential message passing, the same synthetic lesion deficit analysis could be a useful way of finding further points of contact between alternative framings of the motor control problem. In place of looking for similarities in behaviour between lesioned brains and lesioned generative models, we could perform the same comparison between lesioned generative models and disruptions to computational models with a non-inferential framing. However, for this to be possible, it is necessary to demonstrate that both approaches can reproduce the same kind of behaviour. In the next section, we provide a brief illustration that uses the approach in the main text to simulate a paradigm sometimes framed as a specific prediction of the inverse modelling perspective.

Minimum intervention

It is interesting to consider whether there are specific predictions that disambiguate between the presence or absence of the additional (i.e., inverse) model hypothesised under optimal motor control theories. For example, one of the claims made from an optimal motor control perspective is that, when

a trajectory is perturbed by a force, only those perturbations that have relevance for a task will be corrected (McNamee and Wolpert, 2019). A specific example of this is in reaching for a target that may be either a point or a bar. When a force field is applied, greater corrective movements are elicited for the point target as opposed to the bar. This is because a perturbation parallel to the bar does not prevent reaching for (another part of) the target, while it would for a narrower target. While this is sometimes framed as a specific prediction of the optimal motor control hypothesis, the same behaviour is predicted under active inference if our generative model assumed the full length of the bar were equally attractive.

To express the goal of reaching for a wide target through a forward model, we simply project the dynamics predicted by this model onto a direction orthogonal to that target. This induces the belief that the hand is being pulled towards the target, where the attractive location of the target is the point at which a line drawn from the hand to that location is orthogonal to the widest dimension of the target. Figure S2.1 shows the result of simulating this with narrow or wide targets, with or without a disruptive force (introduced one quarter of the way through the trajectory). This force is introduced to the simulated dynamics of the arm that generate the data presented to our synthetic agent, but is not included explicitly in the forward model. It acts to provide an angular acceleration that internally rotates the shoulder joint. The key points to draw from this are that the trajectories for the narrow and wide targets are identical in the absence of a disruptive force, that the trajectory for the narrow target still ends in the same location in the presence of the force, and that the trajectory for the wide target is not corrected to account for the force. This implies the correction only of 'task relevant' perturbations.

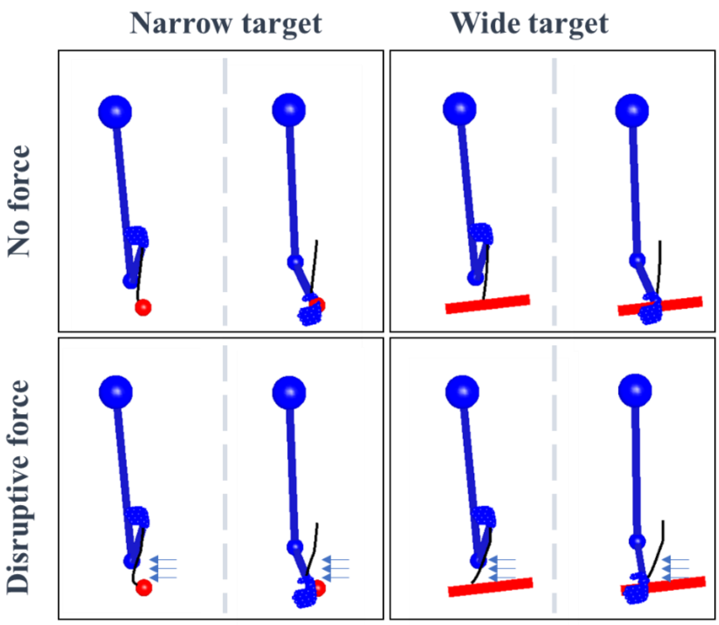

Figure S2.1 – Minimum intervention under active inference.

This Figure shows the trajectory of an arm during a reaching task under four different conditions. Each graphic shows the arm at the start and end of a short trajectory (shown in black). In the upper two boxes, no external perturbation was applied. For the lower two, we introduced an external force. After the first (temporal) quarter of the trajectory, we applied a constant force which, unopposed, would lead to internal rotation at the shoulder joint (indicated by three blue arrows). The key thing to draw from this figure is that the effects of this disruptive force are only compensated for in the 'narrow target' condition. When the target is wider, there is no need to compensate for the deviation caused by the force, as a different part of the target may still be reached.

Crucially, we have produced these simulation results in the absence of an inverse model. Although the same predicted data can also be generated with inclusion of inverse modelling, our approach effectively makes an inverse model (and the hypothesis associated with it) redundant and therefore constitutes a simpler model (which would be favoured by Occam's principle).

Above, we alluded to the importance of using a belief-based scheme. This is something we have not fully exploited in the current paper, but which may yield one of the key predictions that could disambiguate between the two approaches. Under a belief-based scheme, where the prior over policies may be defined in terms of current beliefs, it becomes possible to select policies that maximise the information gain about unknown parameters or states of the generative model. This facilitates explorative behaviour that enables active inference and active learning (Friston *et al.*, 2016). The relevance of this might be in learning the generative model we have employed here, for example, during development. This implies a more directed form of motor babbling, in which actions are selected not at random, but based upon how they inform beliefs about the working of the arm. The prediction here is that learning of this model should be faster than with random action choices, or those based upon a static value function.

Supplementary appendix 3 – Generalised fluctuations

This appendix follows the approach of (Cox and Miller, 1965; Friston *et al.*, 2010), and illustrates an approach to specifying the precision matrix ($\tilde{\Pi} = \tilde{\Sigma}^{-1}$) associated with a variable specified in generalised coordinates of motion. While a little technical, this is important to spell out explicitly, given the emphasis in the main text on a possible role of the cerebellum in optimising these precisions. This specification rests upon a Taylor series expansion of a local trajectory:

$$\begin{aligned} x(t) &\approx x(\tau) + \Delta\tau\, x'(t)\big|_{t=\tau} + \frac{1}{2}\Delta\tau^2 x''(t)\big|_{t=\tau} + ... \\ \Delta\tau &= t - \tau \end{aligned} \quad \text{(S3.1}$$

The coefficients of the Taylor series are the position, velocity, acceleration, etc. of the variable. The rate of change of each of these are given by a deterministic function (*f*) and stochastic fluctuations (ω):

$$\begin{aligned} \dot{\tilde{x}} &= \tilde{f}(\tilde{x}) + \tilde{\omega} \\ \tilde{\omega} &\triangleq \begin{bmatrix} \omega \\ \omega' \\ \omega'' \\ \omega''' \\ \vdots \end{bmatrix} = \begin{bmatrix} \omega^{[0]} \\ \omega^{[1]} \\ \omega^{[2]} \\ \omega^{[3]} \\ \vdots \end{bmatrix} \end{aligned} \quad \text{(S3.2}$$

The random fluctuations may be characterised as follows:

$$\begin{aligned} p(\tilde{\omega}) &= \mathcal{N}(0, \tilde{\Pi}) \\ E[\omega^{[0]}(\tau)] &= 0 \\ E[\omega^{[0]}(\tau) \cdot \omega^{[0]}(\tau)] &= \Sigma \end{aligned} \qquad \text{(S3.3}$$

The autocorrelation function with lag (h) is:

$$\rho(h) \triangleq \tfrac{1}{\Sigma} \underbrace{E[\omega^{[0]}(\tau) \cdot \omega^{[0]}(\tau + h)]}_{\text{Covariance}} \qquad \text{(S3.4}$$

This means the covariance between the fluctuations at two time-points may be factorised into an autocorrelation and a variance. We define the ith derivative of the random fluctuations as:

$$\begin{aligned} \omega^{[i]}(\tau) &\triangleq \lim_{\Delta\tau \to 0} \{\omega^{[i]}(\tau, \Delta\tau)\} \\ \omega^{[i]}(\tau, \Delta\tau) &\triangleq \frac{\omega^{[i-1]}(\tau + \Delta\tau) - \omega^{[i-1]}(\tau)}{\Delta\tau} \end{aligned} \qquad \text{(S3.5}$$

This affords an expression of the covariance between two pairs of coordinates of generalised motion. The covariance between the position and velocity can be found as follows:

$$\begin{aligned} & E[\omega^{[1]}(\tau, \Delta\tau) \cdot \omega^{[0]}(\tau + h)] \\ &= \tfrac{1}{\Delta\tau} E\left[\left(\omega^{[0]}(\tau + \Delta\tau) - \omega^{[0]}(\tau)\right) \omega^{[0]}(\tau + h)\right] \\ &= \tfrac{1}{\Delta\tau} \Sigma\left(\rho(h - \Delta\tau) - \rho(h)\right) \\ \\ & E[\omega^{[1]}(\tau) \cdot \omega^{[0]}(\tau + h)] \\ &= \lim_{\Delta\tau \to 0} \{\tfrac{1}{\Delta\tau} \Sigma\left(\rho(h - \Delta\tau) - \rho(h)\right)\} \\ &= \Sigma \dot{\rho}(h) \end{aligned} \qquad \text{(S3.6}$$

For subsequent derivatives, we get:

$$E[\omega^{[n]}(\tau)\cdot\omega^{[m]}(\tau+h)] = (-1)^{nm}\Sigma\rho^{[m+n]}(h) \Rightarrow$$

(S3.7

$$\tilde{\Pi} = \Sigma^{-1}\otimes\begin{bmatrix}\rho(0) & 0 & \ddot{\rho}(0) & \\ 0 & -\ddot{\rho}(0) & 0 & \\ \ddot{\rho}(0) & 0 & \ddot{\ddot{\rho}}(0) & \\ & & & \ddots\end{bmatrix}^{-1} = \Pi\otimes S(\lambda)$$

The advantage of this expression is that it expresses the covariance structure for an arbitrary temporal embedding order. In terms of the Taylor series approximation of Equation S3.1, this lets us go past the linear and even quadratic terms of the expansion and provides a principled way of defining the precision for a trajectory expressed in terms of all these coefficients. Choosing the autocorrelation function to be Gaussian, we have:

$$\begin{aligned}
\rho(h) &= e^{-\frac{1}{2}\lambda^{-1}h^2} & \rho(0) &= 1 \\
\dot{\rho}(h) &= -\lambda^{-1}h\rho(h) & \dot{\rho}(0) &= 0 \\
\ddot{\rho}(h) &= \lambda^{-1}(\lambda^{-1}h^2-1)\rho(h) & \ddot{\rho}(0) &= -\lambda^{-1} \\
\dddot{\rho}(h) &= \lambda^{-2}h(\lambda^{-1}h^2-3)\rho(h) & \dddot{\rho}(0) &= 0 \\
\ddddot{\rho}(h) &= \lambda^{-2}(\lambda^{-2}h^4-6\lambda^{-1}h^2+3)\rho(h) & \ddddot{\rho}(0) &= 3\lambda^{-2}
\end{aligned}$$

(S3.8

The variance term (λ) can be thought of as parameterising the smoothness of the random fluctuations. This may itself be optimised in relation to data through minimisation of free energy.

References

Adams RA, Shipp S, Friston KJ. Predictions not commands: active inference in the motor system. Brain Structure & Function 2013; 218(3): 611-43.

Baltieri M, Buckley LC. PID Control as a Process of Active Inference with Linear Generative Models. Entropy 2019; 21(3).

Bastos Andre M, Usrey WM, Adams Rick A, Mangun George R, Fries P, Friston Karl J. Canonical Microcircuits for Predictive Coding. Neuron 2012; 76(4): 695-711.

Beal MJ. Variational algorithms for approximate Bayesian inference. University of London United Kingdom; 2003.

Blakemore S-J, Wolpert D, Frith C. Why can't you tickle yourself? NeuroReport 2000; 11(11): R11-R6.

Brown H, Adams RA, Parees I, Edwards M, Friston K. Active inference, sensory attenuation and illusions. Cogn Process 2013; 14(4): 411-27.

Brown H, Friston KJ. Free-Energy and Illusions: The Cornsweet Effect. Frontiers in Psychology 2012; 3: 43.

Cox DR, Miller HD. The theory of stochastic processes. 1965.

Dayan P, Hinton GE, Neal RM, Zemel RS. The Helmholtz machine. Neural computation 1995; 7(5): 889-904.

Egger SW, Remington ED, Chang C-J, Jazayeri M. Internal models of sensorimotor integration regulate cortical dynamics. Nature Neuroscience 2019; 22(11): 1871-82.

Feldman AG, Levin MF. The Equilibrium-Point Hypothesis – Past, Present and Future. In: Sternad D, editor. Progress in Motor Control: A Multidisciplinary Perspective. Boston, MA: Springer US; 2009. p. 699-726.
Feldman H, Friston K. Attention, Uncertainty, and Free-Energy. Frontiers in Human Neuroscience 2010; 4(215).
Friston K. What Is Optimal about Motor Control? Neuron 2011; 72(3): 488-98.
Friston K, FitzGerald T, Rigoli F, Schwartenbeck P, O'Doherty J, Pezzulo G. Active inference and learning. Neuroscience & Biobehavioral Reviews 2016; 68: 862-79.
Friston K, FitzGerald T, Rigoli F, Schwartenbeck P, Pezzulo G. Active Inference: A Process Theory. Neural Comput 2017a; 29(1): 1-49.
Friston K, Mattout J, Kilner J. Action understanding and active inference. Biological cybernetics 2011; 104(1-2): 137-60.
Friston K, Stephan K, Li B, Daunizeau J. Generalised filtering. Mathematical Problems in Engineering 2010; 2010.
Friston KJ, Frith CD. Active inference, communication and hermeneutics(). Cortex; a Journal Devoted to the Study of the Nervous System and Behavior 2015; 68: 129-43.
Friston KJ, Parr T, Vries Bd. The graphical brain: belief propagation and active inference. Network Neuroscience 2017b; 0(ja): 1-78.
Friston KJ, Rosch R, Parr T, Price C, Bowman H. Deep temporal models and active inference. Neuroscience & Biobehavioral Reviews 2017c; 77: 388-402.
Loeliger HA, Dauwels J, Hu J, Korl S, Ping L, Kschischang FR. The Factor Graph Approach to Model-Based Signal Processing. Proceedings of the IEEE 2007; 95(6): 1295-322.
McNamee D, Wolpert DM. Internal Models in Biological Control. Annual Review of Control, Robotics, and Autonomous Systems 2019; 2(1): 339-64.
Parr T, Friston KJ. The Discrete and Continuous Brain: From Decisions to Movement—and Back Again. Neural computation 2018(30): 2319–47.
Parr T, Markovic D, Kiebel SJ, Friston KJ. Neuronal message passing using Mean-field, Bethe, and Marginal approximations. Scientific reports 2019; 9(1): 1889.
Perrinet LU, Adams RA, Friston KJ. Active inference, eye movements and oculomotor delays. Biological cybernetics 2014; 108(6): 777-801.
Shipp S, Adams RA, Friston KJ. Reflections on agranular architecture: predictive coding in the motor cortex. Trends in Neurosciences 2013; 36(12): 706-16.
Yedidia JS, Freeman WT, Weiss Y. Constructing free-energy approximations and generalized belief propagation algorithms. IEEE Transactions on Information Theory 2005; 51(7): 2282-312.
